# Caspase-1-dependent inflammasomes mediate photoreceptor cell death in photo-oxidative damage-induced retinal degeneration

**DOI:** 10.1038/s41598-020-58849-z

**Published:** 2020-02-10

**Authors:** Yvette Wooff, Nilisha Fernando, Josephine H. C. Wong, Catherine Dietrich, Riemke Aggio-Bruce, Joshua A. Chu-Tan, Avril A. B. Robertson, Sarah L. Doyle, Si Ming Man, Riccardo Natoli

**Affiliations:** 1 The John Curtin School of Medical Research, The Australian National University, Canberra, ACT Australia; 2 The ANU Medical School, The Australian National University, Canberra, ACT Australia; 3 School of Chemistry and Molecular Bioscience, The University of Queensland, St. Lucia, QLD 4072 Australia; 4 Department of Clinical Medicine, School of Medicine, Trinity College Institute of Neuroscience, Trinity College, Dublin, Ireland; 5 The National Children’s Research Centre, Our Lady’s Children’s Hospital Crumlin, Dublin, Ireland

**Keywords:** Diseases, Macular degeneration

## Abstract

Activation of the inflammasome is involved in the progression of retinal degenerative diseases, in particular, in the pathogenesis of Age-Related Macular Degeneration (AMD), with NLRP3 activation the focus of many investigations. In this study, we used genetic and pharmacological approaches to explore the role of the inflammasome in a mouse model of retinal degeneration. We identify that *Casp1/11*^*−/−*^ mice have better-preserved retinal function, reduced inflammation and increased photoreceptor survivability. While *Nlrp3*^*−*/*−*^ mice display some level of preservation of retinal function compared to controls, pharmacological inhibition of NLRP3 did not protect against photoreceptor cell death. Further, *Aim2*^*−/−*^, *Nlrc4*^*−/−*^, *Asc*^*−/−*^, and *Casp11*^*−/−*^ mice show no substantial retinal protection. We propose that CASP-1-associated photoreceptor cell death occurs largely independently of NLRP3 and other established inflammasome sensor proteins, or that inhibition of a single sensor is not sufficient to repress the inflammatory cascade. Therapeutic targeting of CASP-1 may offer a more promising avenue to delay the progression of retinal degenerations.

## Introduction

Age-Related Macular Degeneration (AMD) is a chronic inflammatory disease that is characterised by central vision loss due to retinal pigmented epithelium (RPE) and photoreceptor cell death in the macular region of the retina^1,2^. While environmental, lifestyle and genetic risk factors are well established^2–4^, it is increasingly clear that central to disease progression is the accumulation of oxidative stress^5^ and inflammation^6^.

A central component of inflammation is the inflammasome, multi-protein oligomers which form part of the innate immune system, acting in the first line of defence, sensing pathogen-derived or danger signals from pathogen invasion, or cellular stress signals including extracellular ATP or host dsDNA^1,7–9^. Inflammasomes are composed of a pattern recognition receptor (PRR) sensor protein, including NACHT, LRR and PYD domains-containing protein 1 (NLRP1), NLRP3, NAIP-NLRC4, Absent in Melanoma 2 (AIM2), and Pyrin. Following activation, these sensor proteins form a complex with the adaptor protein Apoptosis-associated speck-like protein containing a CARD (ASC, or PYCARD) and protease enzyme Caspase-1 (CASP-1)^10,11^. Activated CASP-1 in turn, cleaves and activates pro-inflammatory cytokines including interleukin-1β (IL-1β), large amounts of which are known to cause microglial activation and macrophage recruitment from the periphery, and ultimately photoreceptor cell death, characteristic features of AMD pathogenesis^3,8,12–16^. Inhibition of IL-1β has been shown to reduce inflammation and further cell death^14^, highlighting the key role of the inflammasome in the progression of retinal degenerative diseases such as AMD.

The most widely studied inflammasome receptor protein, NLRP3, has been postulated to contribute to the progression of AMD^17–23^. NLRP3 can be activated by a large variety of stimuli, many of which are known to be upregulated in AMD pathogenesis, such as mitochondrial reactive oxygen species generation^24^, potassium efflux^25^, and ATP. In addition, NLRP3 can be activated by stimuli implicated in disease progression including complement components C1q^21^, C3a, and membrane attack complex^17,26^, or lipofuscin component bisretinoid A2E and amyloid beta both of which have been isolated in drusen deposits of patients with AMD^27–29^. For these reasons NLRP3 could be a prime candidate for propagating inflammation in AMD, however, extensive research into the role of NLRP3 to date has not shown a definitive role for this inflammasome in disease development^17,30^.

Several studies have been conducted to investigate if and how NLRP3 may be activated in AMD disease progression^21,27–29,31–36^. However, to date studies using animal models have been focused on wet-AMD^21^, while those which investigate the role of NLRP3 in dry-AMD pathogenesis have been largely cell culture-based and focused on the RPE^19,21,22,27,28,31,32^. Although they demonstrate that the NLRP3 inflammasome can be activated in RPE by various stimulations such as oxidative stress^19^, accumulation of repetitive transposable elements of non-coding RNA (*Alu* RNA)^22,32^, or inflammatory pathway stimulants including drusen components lipofuscin^31^, C1q^21^, or Aβ-peptide 1–40^27,28^; they do not provide any conclusive evidence to show NLRP3 involvement in AMD disease progression. In addition, a recent review of commercially available NLRP3 antibodies used in these studies has demonstrated the poor specificity and reliability of these products, finding that no studies showing evidence of NLRP3 involvement in AMD, or its presence in primary or established RPE cell lines, could be replicated^30^. This review, along with evidence implicating a negative role for NLRP3 in *Alu*-RNA induced retinal degeneration^22,32^, and a positive role in laser-induced choroidal neovascularisation (CNV)^21^, highlights the need for *in vivo* investigations into the role of the NLRP3 inflammasome, specifically avoiding the primary use of cell culture-based systems.

Furthermore, little has been studied on the role of other inflammasome pathways in the progression of retinal degenerations, including NLRC4 and AIM2^37,38^ (and reviewed in^39^), or downstream inflammasome components ASC, Caspase-11 (CASP-11), or CASP-1^40–42^. CASP-1 is the effector protein for multiple inflammasome complexes, including NLRP1, NLRC4, AIM2, and Pyrin^43,44^, and in addition to its role in the cleavage of IL-1β and IL-18, is involved in the cleavage of the pyroptosis-inducing, pore-forming protein Gasdermin D^44,45^. Investigations into the role of this central inflammatory component are therefore essential in determining the role of inflammasome pathways in retinal degenerations such as AMD.

This study therefore aims to investigate the role of key components of multiple inflammasome pathways using a photo-oxidative damage (PD)-induced model of retinal degeneration that recapitulates key aspects of dry-AMD^46^. Using this rodent model, we have previously shown that exposure to damaging levels of light causes an increase in oxidative stress and inflammation in the retina, initiating pathological changes seen in AMD^46^, including microglial activation and recruitment^47^, complement deposition^48–50^ and focal photoreceptor and RPE cell loss^49^. In the present study, we found that mice lacking CASP-1 and CASP-11 (*Casp1/11*^*−/−*^ mice) have increased photoreceptor survivability, better-preserved retinal function and reduced inflammation following photo-oxidative damage. *Nlrp3*^*−/−*^ but not NLRP3-pharmacologically inhibited mice have some preservation of retinal function following photo-oxidative damage, whereas *Casp11*^*−/−*^, *Aim2*^*−/−*^, *Nlrc4*^*−/−*^ or *Asc*^*−/−*^ mice show no improvement in retinal function or survivability. Our study highlights CASP-1 as an important mechanistic target for reducing inflammasome mediated cell death in retinal degenerations and for therapeutic intervention.

## Results

### *Casp1/11*^*−*/*−*^ mice exhibit better-preserved retinal survivability

**Figure 1:**
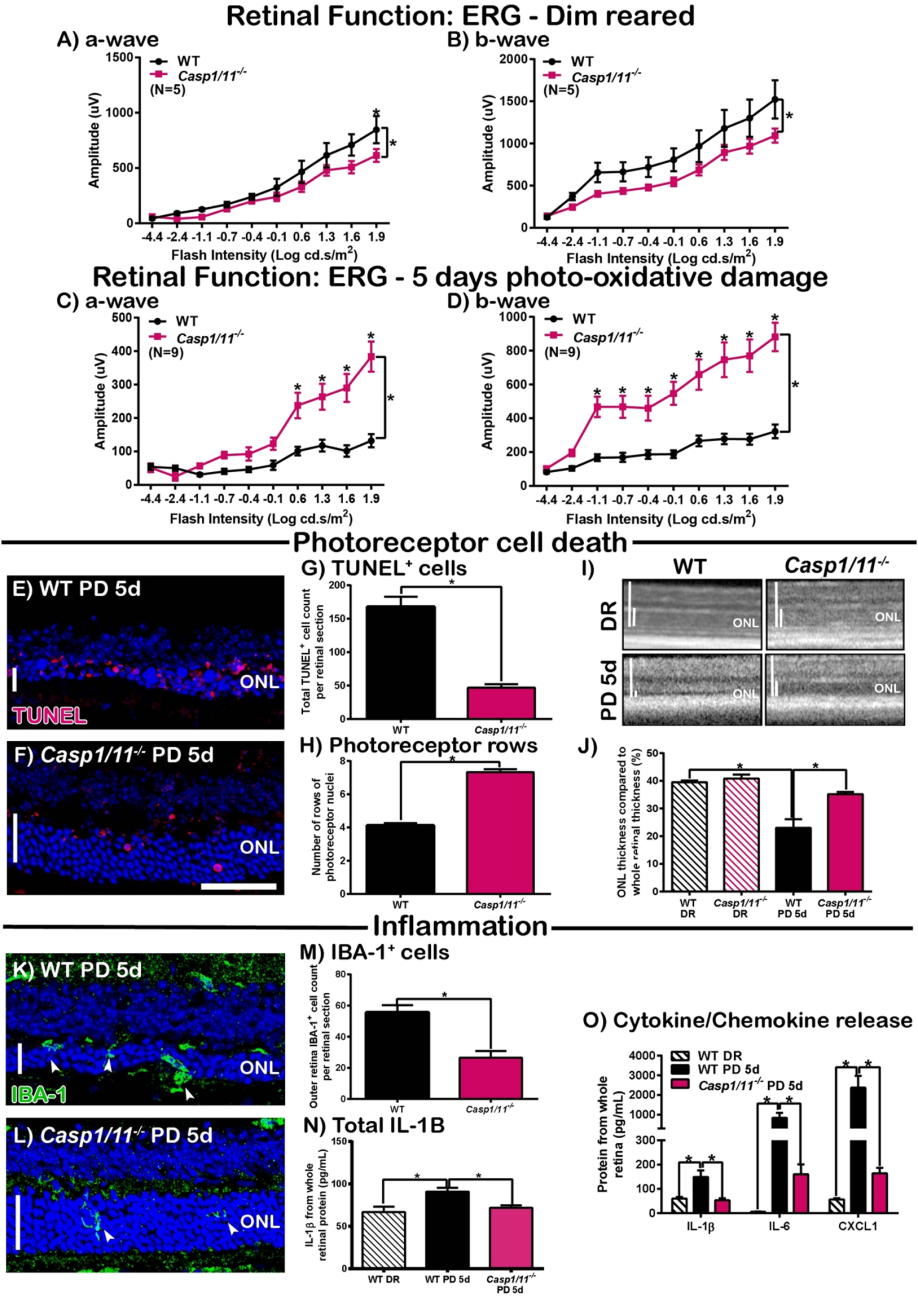
*Caspase1/11*^*−*/*−*^ mice have better-preserved retinal function and reduced inflammation and cell death following photo-oxidative damage (PD) compared to WT controls. (**A**–**D**) Retinal function was measured before (DR) and after 5 days PD using ERG. *Casp1/11*^*−/−*^ mice had significantly lower retinal function in DR conditions compared to WT controls, for both (**A**) a-wave and (**B**) b-wave responses (P < 0.05, N = 6). However, following 5 days PD, retinal function in *Casp1/11*^*−/−*^ mice was significantly higher than WT PD controls for both (**C**) a-wave and (**D**) b-wave response (P < 0.05, N = 10). E-J The effect of *Casp1/11* deficiency on photoreceptor cell death. Representative confocal images show ONL thickness and TUNEL^+^ cells in the ONL of (**E**) WT and (**F**) *Casp1/11*^*−/−*^ mice following PD. (**G**) *Casp1/11*^*−/−*^ mice has significantly fewer TUNEL^+^ cells in the ONL and (**H**) significantly more photoreceptor rows than WT PD controls (P < 0.05, N = 6). (**I**,**J**) There was no significant difference in ONL thickness between DR *Casp1/11*^*−/−*^ mice and WT controls (P > 0.05, N = 6), however, after PD, the ONL of *Casp1/11*^*−/−*^ mice was significantly thicker than WT PD controls (P < 0.05, N = 6). (**K**–**P**) The effect of *Casp1/11* deficiency on inflammation in the retina following PD. Representative confocal images show an increased number of IBA-1^+^ microglia in (**K**) WT PD mice compared to fewer in (**L**) *Casp1/11*^*−/−*^ PD mice. (**M**) *Casp1/11*^*−/−*^ mice had significantly fewer IBA-1^+^ microglia in the outer retina than WT controls following PD (P < 0.05, N = 6). (**N**) *Casp1/11*^*−/−*^ PD mice had a significantly reduced level of IL-1β protein as measured by ELISA than WT PD controls (P < 0.05, N = 6). (**O**) In addition, *Casp1/11*^*−/−*^ PD mice has significantly reduced levels of IL-1β, IL-6 and CXCL1 compared to WT PD mice, (P < 0.05, N = 6). Scale bars = 50 μM. *WT controls used in Fig. 1 are the same as in Fig. 4, with all mice participating in the same experimental run.

To elucidate the contribution of the inflammasome in the progression of retinal degenerations, we used *Casp1/11*^*−/−*^ mice to investigate the role of the inflammasome Caspases, CASP-1 and CASP-11 in the retina. The retinal function of WT and *Casp1/11*^*−/−*^ mice housed in dim-reared conditions and following 5 days photo-oxidative damage was measured using electroretinography (ERG). Dim-reared *Casp1/11*^*−/−*^ mice had significantly lower ERG responses for both a-wave and b-wave measures compared to dim-reared controls (Fig. 1A,B, P < 0.05). However, following photo-oxidative damage, both a-wave and b-wave responses were significantly higher compared to WT photo-oxidative damaged mice (Fig. 1C,D, P < 0.05), demonstrating better-preservation of retinal function. The protected retinal function in *Casp1/11*^*−/−*^ mice was reflected by a significantly decreased number of TUNEL^+^ cells (dead cells) in the outer retina (Fig. 1E–G, P < 0.05), increased photoreceptor row counts (Fig. 1H, P < 0.05), and increased ONL thickness (Fig. 1I,J, P < 0.05) compared to WT controls. In addition, *Casp1/11*^*−/−*^ mice had significantly reduced IBA-1^+^ cell counts, a marker of microglia/macrophage immune cells, in the outer retina (Fig. 1K–M, P < 0.05), and reduced IL-1β protein levels as measured by ELISA and multiplex assays (Fig. 1N,O, P < 0.05). Levels of the cytokine IL-6 and chemokine CXCL1 were also reduced in *Casp1/11*^*−/−*^ mice compared with WT mice (Fig. 1O, P < 0.05). Taken together, these results highlight the key role that the inflammasome plays in mediating inflammatory cell death during retinal degenerative diseases induced by photo-oxidative damage.

### Inflammasome components are upregulated in response to photo-oxidative damage

**Figure 2:**
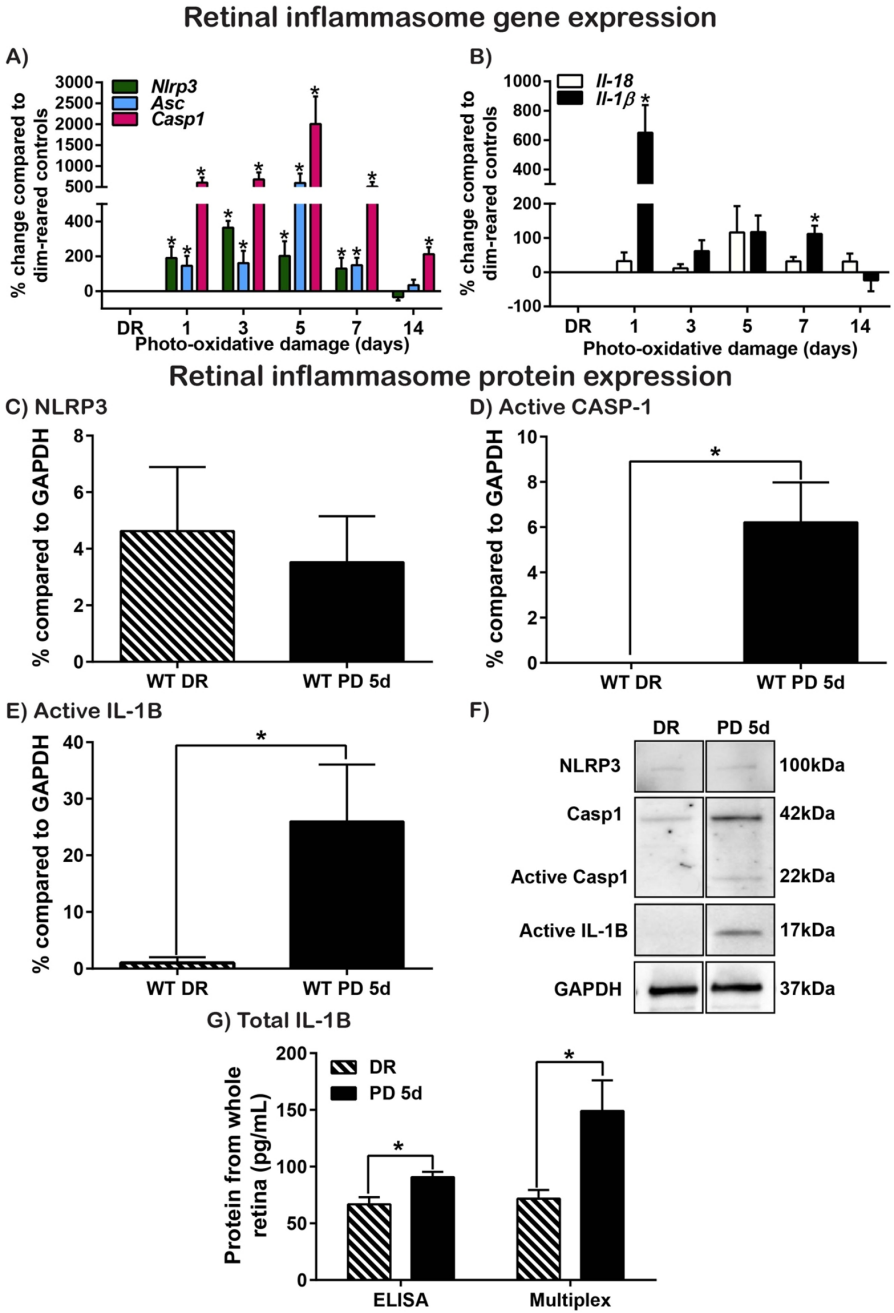
Retinal inflammation over photo-oxidative damage (PD) time course. (**A**,**B**) Inflammasome and inflammatory gene expression measured across a time-course of PD. (**A**) Inflammasome components *Nlrp3*, *Asc* and *Casp-1* increased significantly from 1–7 days PD (P < 0.05). *Nlrp3* expression was highest at 3 days PD while expression of *Asc* and *Casp-1* peaked at 5 days PD. *Casp-1* expression was still significantly increased at 7 days recovery (14 day time point) compared to DR controls (P < 0.05, N = 5–8). (**B**) Pro-inflammatory cytokine gene expression was measured across a time-course of PD, with *Il-1β* expression significantly upregulated at 1 day and 7 days PD (P < 0.05), but not at 3–5 days PD, or recovery (14 days) (P < 0.05). No significant change was detected in *Il-18* expression across the time-course (P > 0.05, N = 5–8). (**C**–**G**) Inflammasome protein expression in the retina following 5 days PD. (**C**) NLRP3 expression was unchanged between DR and PD mice (P > 0.05), however, both (**D**) active CASP-1 and (**E**) active IL-1β were significantly increased at 5 days PD compared to DR controls (P < 0.05). (**F**) Representative, cropped western blots showing bands of 100 kDa for NLRP3, 42 kDa for pro-CASP-1, 22 kDa for active-CASP-1, 17 kDa for active IL-1β and 37 kDa for GAPDH reference protein, in retinal protein lysates from DR and PD retinas. Replicate blots were processed in parallel, and full-length blots are presented in Supplementary Fig. 5. (**G**) Total IL-1β levels were increased in PD retinal lysates compared to DR controls as measured by both ELISA and Multiplex Magnetic Bead Assay (P < 0.05, N = 4–5).

To determine the potential role of the inflammasome in AMD pathogenesis, we used qRT-PCR to compare the gene expression of inflammasome components and secreted pro-inflammatory cytokines in the retina across a time-course of photo-oxidative damage. Inflammasome components *Nlrp3*, *Asc* and *Casp-1* were significantly upregulated from 1–7 days photo-oxidative damage, with *Casp-1* expression remaining significantly elevated at 14 days (7 days photo-oxidative damage +7 days recovery). *Nlrp3* expression was highest at 3 days photo-oxidative damage, while the expression of *Asc* and *Casp-1* reached a peak at 5 days photo-oxidative damage (Fig. 2A, P < 0.05).

The expression pattern of the pro-inflammatory cytokine *Il-1β* was biphasic, being very highly upregulated at 1 day photo-oxidative damage, dropping down at 3 days, and steadily increasing at 5 and 7 days (Fig. 2B, P < 0.05). No change was seen in the expression of *Il-18* over the protracted time-course of photo-oxidative damage (Fig. 2B, P > 0.05). Western blot analysis of inflammasome components showed that while there was no change in the protein levels of NLRP3 between dim-reared and 5 days photo-oxidative damage retinas (Fig. 2C, P > 0.05), there was a significant increase in active CASP-1 (Fig. 2D, P < 0.05) and active IL-1β (Fig. 2E, P < 0.05) following photo-oxidative damage (Fig. 2F). A significant increase in total IL-1β protein levels was detected in retinas of mice with photo-oxidative damage compared to dim-reared controls (Fig. 2G, P < 0.05). These results suggest that inflammasome components are differentially upregulated during retinal degenerations.

### NLRP3 is expressed and localised to inner retinal cells in degeneration

**Figure 3:**
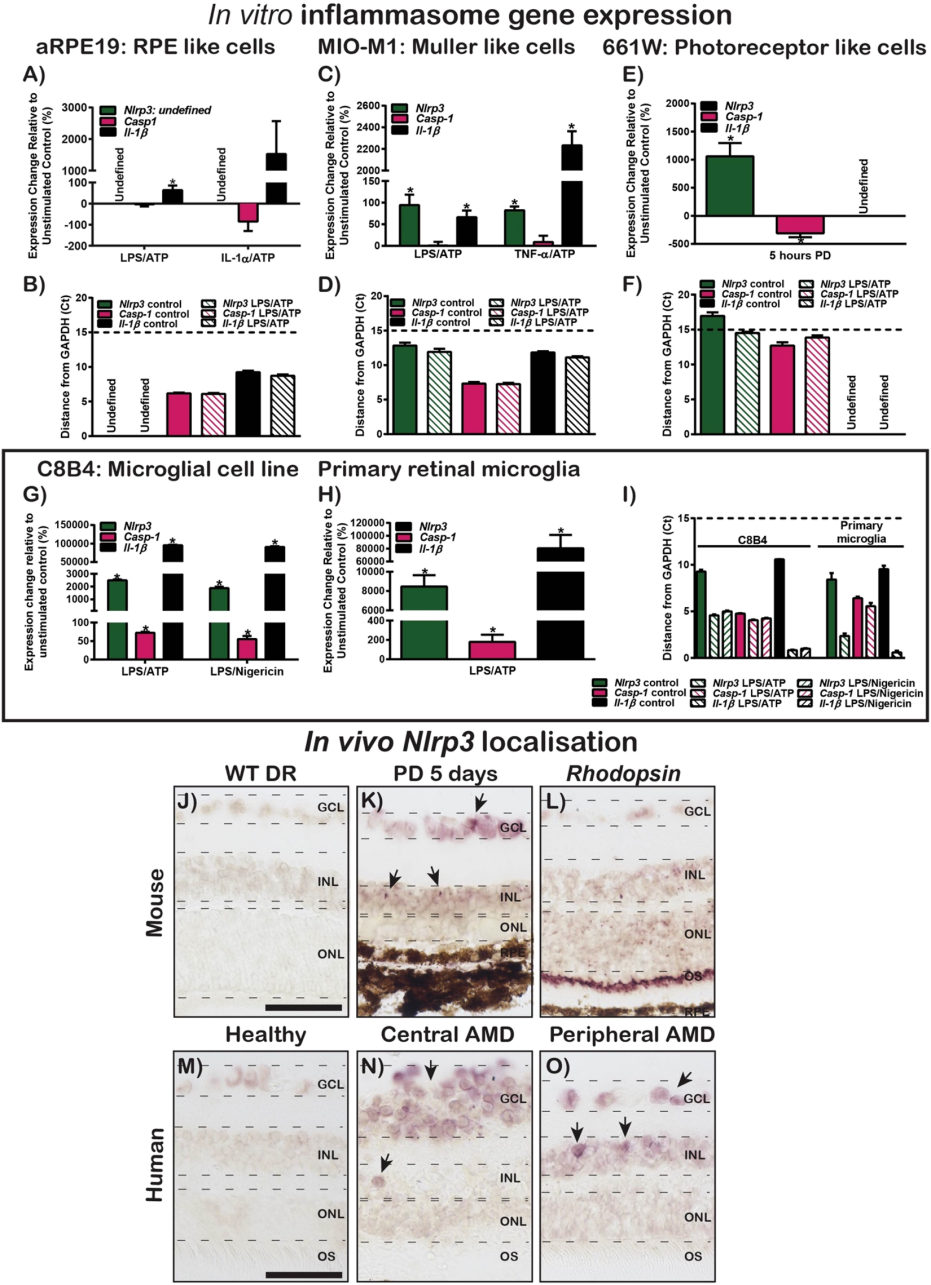
*In vitro* and *in vivo* expression and localisation of inflammasome components. (**A**–**I**) Gene expression and cycle threshold (Ct) of inflammasome components (*Nlrp3*, *Casp-1* and *Il-1β*) following stimulation in *in vitro* cell culture. (**A**) Inflammasome gene expression in aRPE19 cells following stimulation with LPS/ATP or IL-1α/ATP. *Nlrp3* was undetected, *Casp-1* showed no change in expression (P > 0.05, N = 6–8), and *Il-1β* significantly increased following both stimulations (P < 0.05, N = 6–8). (**B**) Ct values for *Casp-1* and *Il-1β* were 6 and 9 cycles from *Gapdh* respectively. (**C**) Inflammasome gene expression in MIO-M1 cells following stimulation with LPS/ATP or TNF-α/ATP. The expression of *Nlrp3* and *Il-1β* significantly increased following both stimulations (P < 0.05, N = 6–8) however, there was no change in the expression of *Casp-1* (P > 0.05, N = 6–8). (**D**) Ct values for *Nlrp3*, *Casp-1* and *Il-1β* were 12, 6 and 11 cycles from *Gapdh* respectively. (**E**) Inflammasome gene expression in 661 W cells following 5 hours bright white light (15k lux) stimulation. *Nlrp3* gene expression significantly increased following stimulation (P < 0.05, N = 6), while *Casp-1* expression decreased significantly (P < 0.05, N = 6), and *Il-1β* was undetected. (**F**) Ct values for *Nlrp3* and *Casp-1* were 15 and 11 cycles from *Gapdh*. (**G**) Inflammasome gene expression in C8B4 cells following stimulation with LPS/ATP or LPS/Nigericin. The expression of *Nlrp3*, *Casp-1* and *Il-1β* all significantly increased following both stimulations (P < 0.05, N = 6). (**H**) Inflammasome gene expression in primary retinal microglia cells following stimulation with LPS/ATP. The expression of *Nlrp3*, *Casp-1* and *Il-1β* all significantly increased following stimulation (P < 0.05, N = 6). (**I**) Ct values for *Nlrp3*, *Casp-1* and *Il-1β* were 6, 6 and 2 cycles respectively from *Gapdh* for stimulated C8B4 cells, and 3, 7 and 1.5 cycles respectively from *Gapdh* for primary microglia following stimulation. (**J**–**O**) Localisation of *Nlrp3* mRNA in mouse and human retinas. (**J**) No expression of *Nlrp3* was seen in WT DR mouse retinas, however, *Nlrp3* was expressed in retinas of (**K**) 5 day PD mice, localising in the INL and GCL. (**L**) *Rhodopsin*, as a positive control, was expressed in the outer segments of photoreceptors. (**M**) In healthy age-matched human donor retinas, no expression of *Nlrp3* was seen, however, *Nlrp3* was found to be expressed in the INL and GCL of human AMD donor retinas in both (**N**) central and (**O**) peripheral sections. Scale bar = 25 μM (Mouse retinal sections), 10 μM (Human retinal sections).

We further investigated the gene expression levels of inflammasome components *Nlrp3*, *Casp-1* and *Il-1β* in RPE (aRPE19), Müller (MIO-M1), photoreceptor (661 W), microglia (C8B4) and primary retinal microglia stimulated with inflammasome activators, to determine which retinal cell types express inflammasome components. Following stimulation of aRPE19 cells with either IL-1α/ATP or LPS/ATP, we observed a lack of expression of *Nlrp3* (Fig. 3A) and no change in the expression of *Casp-1* compared to unstimulated controls (Fig. 3A, P > 0.05). We also observed a small but significant increase in *Il-1β* expression following LPS/ATP stimulation (Fig. 3A,B, P < 0.05). In MIO-M1 cells treated with either LPS/ATP or TNF-α/ATP, the expression levels of *Nlrp3* and *Il-1β* were significantly increased compared with untreated controls (Fig. 3C,D, P < 0.05). However, there was no change in the expression of *Casp-1* following stimulation (Fig. 3C,D, P > 0.05).

To recapitulate photo-oxidative damage, we treated 661 W photoreceptor like-cells for 5 hours with 15 K lux white LED light. Under this condition, the expression of *Nlrp3* was significantly upregulated compared to dim controls (Fig. 3E, P < 0.05), while levels of *Casp-1* decreased significantly (Fig. 3E, P < 0.05). *Il-1β* however, was undetected in both dim and light-treated groups (Fig. 3E). *Nlrp3* gene expression increased significantly following photo-oxidative damage, however, cycle threshold (Ct) values following light exposure were 15 cycles from *Gapdh*, which indicates very low expression of *Nlrp3* in this cell type even following stimulation (Fig. 3F).

C8B4 microglial-like cells treated with either LPS/ATP or LPS/Nigericin showed significantly upregulated expression of *Nlrp3*, *Casp-1* and *Il-1β* (Fig. 3G, P < 0.05). This response was also observed in primary retinal microglia stimulated with LPS/ATP, with the expression of *Nlrp3*, *Casp-1* and *Il-1β* significantly upregulated compared to unstimulated controls (Fig. 3H, P < 0.05). Ct values of *Nlrp3*, *Casp-1* and *Il-1β* in both cell types following inflammasome stimulation were between 5–6, 5–8 and 1–2 cycles from *Gapdh* respectively, indicating very high expression for all inflammasome genes investigated in immortalised and primary retinal microglial cell types (Fig. 3I).

Our results overall indicate that while inflammasome gene expression is inducible to significant levels in retinal immortalised cell types, with the exception of *Nlrp3* in aRPE19 and *Il-1β* in 661 W, the relative expression levels indicate that these components are low-to-moderately expressed in aRPE19 (Fig. 3B), MIO-M1 (Fig. 3D) and 661 W (Fig. 3F) cell lines, and were only highly expressed in both immortalised and primary retinal microglial cell types (Fig. 3I).

The localisation of *Nlrp3* was subsequently investigated in both rodent and human AMD donor retinas using *in situ* hybridisation. No expression of *Nlrp3* was detected in dim-reared controls (Fig. 3J), whereas *Nlrp3* was expressed in the inner nuclear layer (INL) and ganglion cell layer (GCL) following photo-oxidative damage (Fig. 3K). The expression of the positive control gene *Rhodopsin* was localised in the photoreceptor outer segments (Fig. 3L). In healthy age-matched control human donor tissue, no expression of *Nlrp3* was seen in the retina (Fig. 3M), however, *Nlrp3* was expressed in the INL and GCL of both central (Fig. 3N) and peripheral (Fig. 3O) AMD sections.

### *Nlrp3*^*−*/*−*^ mice have better-preserved retinal function after photo-oxidative damage

**Figure 4:**
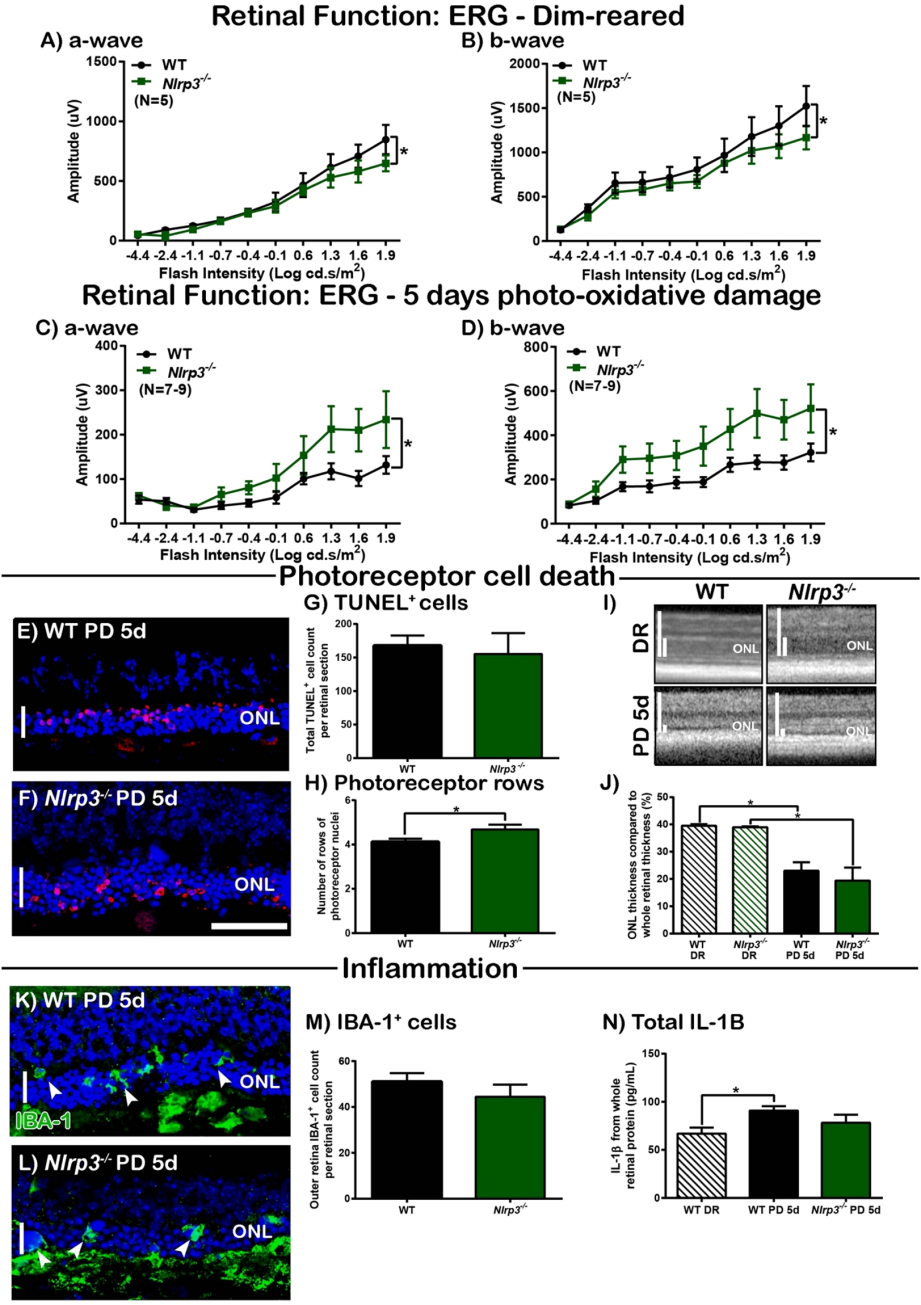
*Nlrp3*^*−*/*−*^ mice show better-preservation of retinal function following PD compared to WT controls. (**A**–**D**) Retinal function was measured before (DR) and after 5 days PD using ERG. *Nlrp3*^*−/−*^ mice had a small but significantly lower retinal function in DR conditions compared to WT controls, for both (**A**) a-wave and (**B**) b-wave (P < 0.05, N = 6). However, following 5 days PD, retinal function in *Nlrp3*^*−/−*^ mice was significantly higher than WT PD controls for both (**C**) a-wave and (**D**) b-wave (P < 0.05, N = 8). (**E**–**J**) The effect of *Nlrp3* deficiency on photoreceptor cell death. Representative confocal images show ONL thickness and TUNEL^+^ cells in the ONL of (**E**) WT PD and (**F**) *Nlrp3*^*−/−*^ PD mice. (**G**) There was no significant difference in TUNEL^+^ cell counts in the ONL between *Nlrp3*^*−/−*^ PD mice and WT controls (P > 0.05, N = 6). (**H**) *Nlrp3*^*−/−*^ PD mice had a small but significant increase in photoreceptor rows than WT controls (P < 0.05, N = 8). (**I**,**J**) There was no significant difference in ONL thickness between *Nlrp3*^*−/−*^ and WT controls for DR or PD groups (P > 0.05, N = 6). (**K**–**P**) The effect of *Nlrp3* deficiency on inflammation in the retina following PD. Representative confocal images show IBA-1^+^ microglia in (**K**) WT PD mice compared to (**L**) *Nlrp3*^*−/−*^ PD mice. (**M**) No significant difference in the number of IBA-1^+^ microglia were seen in the outer retina of *Nlrp3*^*−/−*^ and WT PD mice (P > 0.05, N = 8). (**N**) No change was seen in retinal IL-1β protein levels as measured by ELISA between *Nlrp3*^*−/−*^ and WT PD mice (P > 0.05, N = 6). Scale bars = 50 μM. *WT controls used in Fig. 1 are the same as in Fig. 4, with all mice participating in the same experimental run.

The role of NLRP3, and effects of NLRP3 deficiency on the retina were investigated using *Nlrp3*^*−/−*^ mice. The retinal function of WT and *Nlrp3*^*−/−*^ mice was assessed during dim-reared conditions and after 5 days photo-oxidative damage. Dim-reared *Nlrp3*^*−/−*^ mice had slightly reduced retinal function for both a-wave and b-wave responses compared to WT controls (Fig. 4A,B, P < 0.05), however, following photo-oxidative damage, *Nlrp3*^*−/−*^ mice had significantly higher a- and b-wave retinal responses than WT photo-oxidative damaged mice (Fig. 4C,D, P < 0.05). Photoreceptor survivability was subsequently measured using TUNEL assay, photoreceptor row counts and ONL thickness measurements. We observed no significant difference in total TUNEL^+^ cell counts in the ONL between *Nlrp3*^*−/−*^ and WT mice with photo-oxidative damage (Fig. 4E–G, P > 0.05), and while there was a slight increase in the number of photoreceptor rows in *Nlrp3*^*−/−*^ mice compared to WT controls (Fig. 4H, P < 0.05), ONL thickness measurements from images obtained via Optical Coherence Tomography (OCT) indicated that there was no difference in the thickness of the photoreceptor nuclear layer between these groups (Fig. 4I,J, P > 0.05). Retinal inflammation was also measured in WT and *Nlrp3*^*−/−*^ mice following photo-oxidative damage, however, no change was observed for IBA-1^+^ cell counts in the outer retina (Fig. 4K–M, P > 0.05). In addition, there was no significant change in IL-1β protein levels measured by ELISA (Fig. 4N, P > 0.05).

### NLRP3 inhibition does not protect against photoreceptor cell death

**Figure 5:**
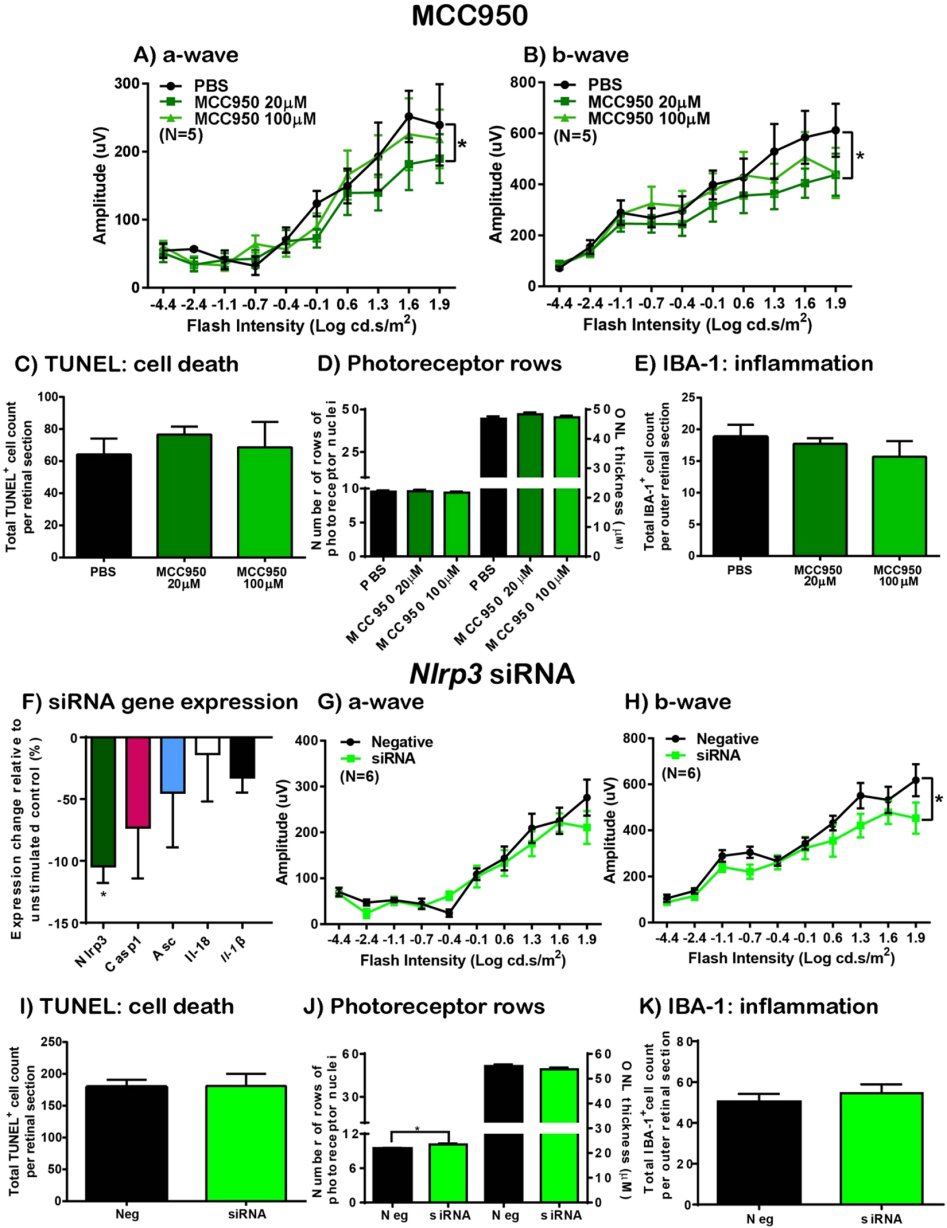
NLRP3 inhibition does not reduce photoreceptor cell death or improve retinal function following PD. (**A**,**B**) Retinal function in mice injected with NLRP3-specific inhibitor MCC950 was measured following 5 days PD using ERG. No change in retinal function was observed between mice injected with 20 µM dose of MCC950 and PBS injected controls for (**A**) a-wave or (**B**) b-wave responses (P > 0.05, N = 5). A dose of 100 µM MCC950 however, resulted in significantly reduced retinal function for both (A) a-wave and (B) b-wave responses compared to PBS injected controls (P < 0.05, N = 5). (**C**–**E**) The effect of MCC950 on retinal cell death and inflammation following 5 days PD. (**C**) No change was seen in TUNEL^+^ cells in the ONL, (**D**) photoreceptor row counts or ONL thickness measurements, or (**E**) IBA-1^+^ cell counts for either 20 µM or 100 µM doses of MCC950 compared to PBS controls (P > 0.05, N = 5). (**F**) *Nlrp3*, but not *Casp-1*, *Asc*, *Il-18* or *Il-1β* gene expression was significantly decreased in siRNA-injected retinas following 5 days PD (P < 0.05, N = 6). (**G**,**H**) Retinal function in mice injected with *Nlrp3* siRNA was measured following 5 days PD using ERG. No change was seen in retinal response between siRNA and negative control group for (**G**) a-wave however, (**H**) there was a significant reduction in retinal function for siRNA group compared to negative control for b-wave. (**I**–**K**) The effect of *Nlrp3* siRNA on the retina following 5 days PD. Compared to controls, in *Nlrp3* siRNA-injected mice, there was no change in (**I**) TUNEL^+^ cells in the ONL (P > 0.05, N = 6), (**J**) a small significant increase in photoreceptor rows (P > 0.05, N = 6), but no change in ONL thickness measurements (P > 0.05, N = 6), and no change in IBA-1^+^ cell counts in the outer retina (P > 0.05, N = 6).

To examine the effects of NLRP3 inhibition on retinal function, inflammation and photoreceptor survivability after photo-oxidative damage; NLRP3 was inhibited by intravitreal injections of MCC950, a specific NLRP3 inhibitor, and siRNA for *Nlrp3*. Compared to PBS injected controls, retinal function for both a- and b-wave measures was significantly reduced after photo-oxidative damage in mice injected with 20 µM MCC950 (Fig. 5A,B, P < 0.05), but was unchanged in mice injected with 100 µM MCC950 (P > 0.05). There was no change in TUNEL^+^ cell counts, photoreceptor row counts, ONL thickness measurements, or IBA-1^+^ cell counts in either dosage group compared to PBS controls (Fig. 5C–E, P < 0.05). Similar results were observed in mice injected with *Nlrp3* siRNA compared to negative control siRNA. After 5 days photo-oxidative damage, *Nlrp3* gene expression was significantly reduced in the retina in mice injected with *Nlrp3* siRNA compared to those injected with negative control siRNA (Fig. 5F, P < 0.05), however, there was no significant change in the expression of downstream inflammasome genes *Casp-1*, *Asc*, *Il-18*, and *Il-1β* (Fig. 5F, P > 0.05). No change was seen in a-wave ERG function (Fig. 5G, P > 0.05), while b-wave function was significantly reduced (Fig. 5H, P < 0.05) compared to controls. In addition, there was no change in TUNEL^+^ cell counts (Fig. 5I, P > 0.05), a small significant increase in photoreceptor row counts (Fig. 5J, P < 0.05), but not ONL thickness measurements (Fig. 5J, P > 0.05), and no change in IBA-1^+^ cell counts in *Nlrp3* siRNA-injected mice (Fig. 5K, P < 0.05) compared to controls. Therefore, pharmacological or siRNA-mediated inhibition of NLRP3 conferred no retinal protection against photo-oxidative damage.

### *Casp11*^*−/−*^ mice show no retinal protection following photo-oxidative damage

**Figure 6:**
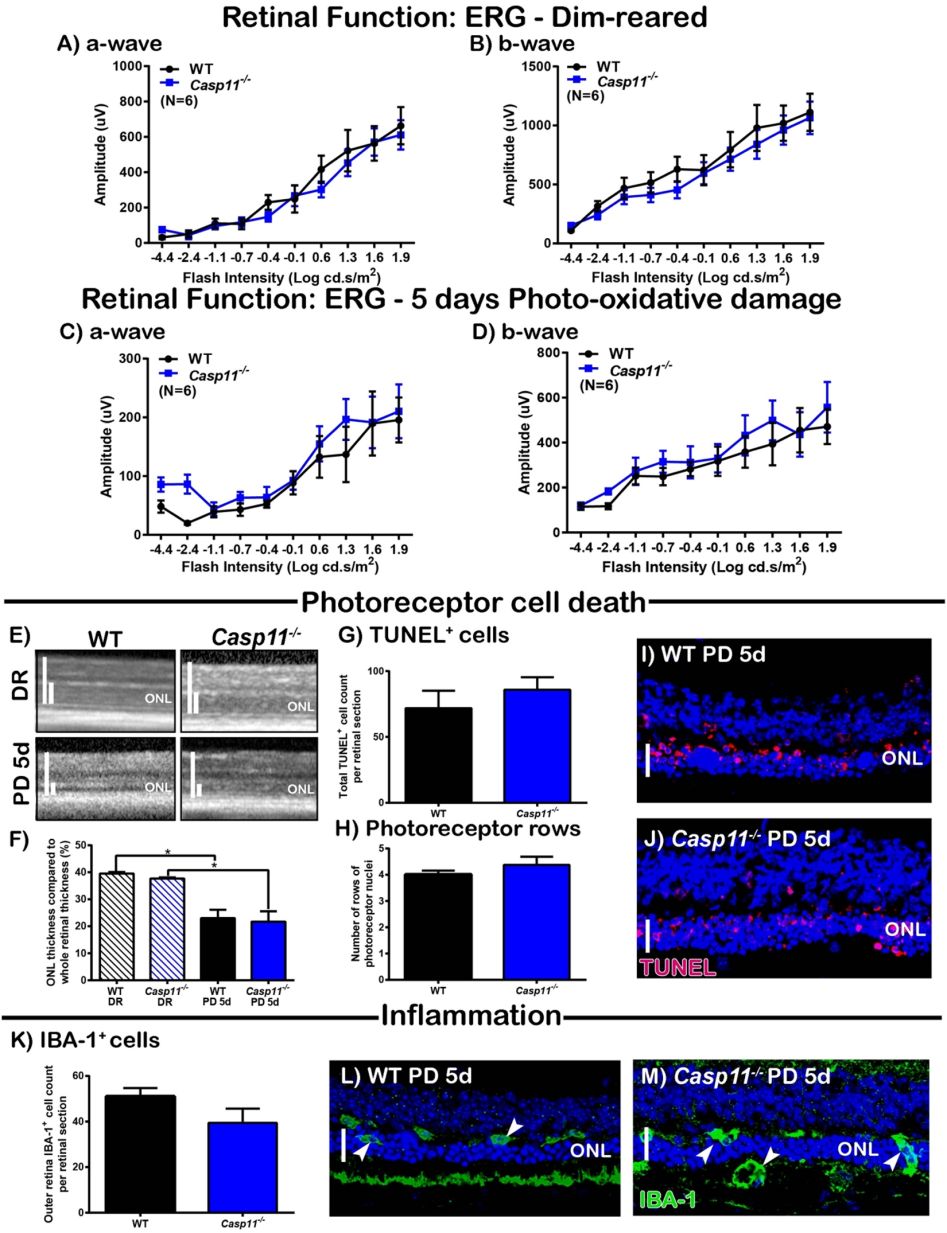
*Casp11*^*−/−*^ mice show no preservation of retinal function following PD. (**A**–**D**) Retinal function was measured before (DR) and after 5 days PD using ERG. There was no significant difference in retinal function between *Casp11*^*−/−*^ and WT mice, in DR conditions for (**A**) a-wave or (**B**) b-wave responses or for (**C**) a-wave or (**D**) b-wave responses following PD (P > 0.05, N = 6). (**E**–**J**) The effect of *Casp-11* deficiency on photoreceptor cell death. (**E**,**F**) No significant difference in ONL thickness was seen between *Casp11*^*−/−*^ and WT mice for DR or PD groups (P > 0.05, N = 6). No significant difference was seen between *Casp11*^*−/−*^ and WT PD groups for (**G**) TUNEL^+^ cell counts in the outer retina or in (**H**) photoreceptor row counts (P > 0.05, N = 6). Representative images show retinal thickness and TUNEL^+^ cells in the ONL for (**I**) WT PD mice and (**J**) *Casp11*^*−/−*^ PD mice. (**K**,**L**) The effect of *Casp-11* deficiency on inflammation in the outer retina following PD. (**K**) No significant difference was seen in total IBA-1^+^ cell counts in the outer retina following PD between *Casp11*^*−/−*^ and WT mice (P > 0.05, N = 6). Representative confocal images show IBA-1^+^ microglia in the outer retina of (**L**) WT PD and (**M**) *Casp11*^*−/−*^ mice. Scale bars = 50 μM.

Given the better-preserved function and survivability of the retina demonstrated by *Casp1/11*^*−/−*^ mice compared to WT controls, the role of the non-canonical pathway of NLRP3 inflammasome activation was investigated via the use of *Casp11*^*−/−*^ mice. No change in retinal function measured by ERG was observed between WT and *Casp11*^*−/−*^ mice for either dim-reared or photo-oxidative damage groups (Fig. 6A–D, P > 0.05). In addition, there was no difference in TUNEL^+^ cell counts, photoreceptor row counts or outer nuclear layer (ONL) thickness measurements between these groups (Fig. 6E–J, P > 0.05). Further, there was no difference in IBA-1^+^ cell counts in the outer retina between WT and *Casp11*^*−/−*^ mice following photo-oxidative damage (Fig. 6K–M, P > 0.05). These results suggest that CASP-1, but not CASP-11, is the likely candidate which mediates retinal cell death and inflammation in our model of retinal degenerative disease.

### *Aim2*^*−/−*^ and *Asc*^*−/−*^ mice, but not *Nlrc4*^*−/−*^ mice have reduced retinal function after photo-oxidative damage

To identify whether inflammasome components in addition to CASP-1 might be responsible for the progression of retinal degenerations, we tested mice lacking NLRC4, AIM2 or ASC in our model of retinal degeneration. Retinal function was significantly decreased between dim-reared WT and *Nlrc4*^*−/−*^ mice (Supplementary Fig. 1A,B, P < 0.05), however, no significant change was observed between WT and *Nlrc4*^*−/−*^ mice following photo-oxidative damage (Supplementary Fig. 1C,D, P > 0.05). No significant change was measured in TUNEL^+^ cell counts in the ONL (Supplementary Fig. 1E,H,I, P > 0.05), however, there was a significant increase in photoreceptor row counts and ONL thickness measurements (Supplementary Fig. 1F, P < 0.05), and a decrease in the number of total IBA-1^+^ cells in the outer retina (Supplementary Fig. 1G,J,K, P < 0.05), compared to WT controls with photo-oxidative damage. Overall these results suggest that NLRC4 does play a major role in mediating retinal cell death in retinal degeneration induced by photo-oxidative damage.

We observed no change in retinal function between dim-reared WT and *Aim2*^*−/−*^ mice (Supplementary Fig. 2A,B, P > 0.05), however, after 5 days photo-oxidative damage, retinal function was significantly reduced in *Aim2*^*−/−*^ mice compared to WT controls (Supplementary Fig. 2C,D, P < 0.05). Furthermore, *Aim2*^*−/−*^ mice had significantly higher levels of cell death as measured by total TUNEL^+^ cell counts in the ONL (Supplementary Fig. 2E,H,I, P < 0.05), and a reduced number of photoreceptor rows (Supplementary Fig. 2F, P < 0.05). However, ONL thickness measurements showed no significant difference compared to WT photo-oxidative damage mice (Supplementary Fig. 2F, P > 0.05). *Aim2*^*−/−*^ mice had reduced IBA-1^+^ cell counts in the outer retina compared to WT mice with photo-oxidative damage (Supplementary Fig. 2G,J,K, P < 0.05).

Finally, we investigated the role of the inflammasome adaptor ASC in retinal degeneration. *Asc*^*−/−*^ mice had reduced ERG function in both dim-reared and photo-oxidative damage conditions compared to WT controls (Supplementary Fig. 3A–D, P < 0.05). Similar to *Aim2*^*−/−*^ mice, following 5 days photo-oxidative damage, *Asc*^*−/−*^ mice had significantly higher levels of cell death as measured by total TUNEL^+^ cell counts in the ONL (Supplementary Fig. 3E,H,I, P < 0.05), a reduced number of photoreceptor rows, as well as reduced ONL thickness (Supplementary Fig. 3F, P < 0.05), and reduced IBA-1^+^ cell counts in the outer retina (Supplementary Fig. 3G,J,K, P < 0.05), compared to WT mice. Taken together, these results suggest a possible protective role for both adaptor protein ASC as well as AIM2 inflammasome sensor protein in retinal degenerations.

### CASP-1 may be required for normal photoreceptor development in the mouse retina

Given the reduction in retinal function of dim-reared *Casp1/11*^*−/−*^ mice compared to WT controls, the length of photoreceptor inner and outer segments was measured on retinal cryosections stained with hematoxylin and eosin. While there was no difference in the length of photoreceptor inner segments between WT and *Casp1/11*^*−/−*^ dim-reared mice, the outer segments were significantly shorter compared to WT controls (Supplementary Fig. 4A–C, P < 0.05). Following 5 days of photo-oxidative damage however, *Casp1/11*^*−/−*^ mice had significantly longer photoreceptor inner and outer segment lengths (measured as combined length due to the condensed and distorted nature of the outer retina in WT mice following photo-oxidative damage), (Supplementary Fig. 4D–F, P < 0.05). These results indicate that CASP-1 may be required for the normal development of retinal photoreceptor outer segments, but following activation of the inflammasome in retinal degenerations, plays a role in mediating photoreceptor cell death.

## Discussion

Results from this study demonstrate an important function of CASP-1 inflammasomes in mediating retinal degenerations, with *Casp1/11*^*−/−*^ mice having significantly better-preserved retinal function, increased photoreceptor survivability, and decreased inflammation compared to controls. We showed that while *Nlrp3*^*−/−*^ mice had some preservation of retinal function following photo-oxidative damage, WT mice injected intravitreally with either *Nlrp3* siRNA or specific inhibitor MCC950, showed decreased or unchanged retinal function compared to controls. Further, *Casp11*^*−/−*^, *Nlrc4*^*−/−*^, *Aim2*^*−/−*^, and *Asc*^*−/−*^ mice showed either a decreased functionality or no change in response to photo-oxidative damage. We reasoned that inflammasome mediated-inflammation in retinal degenerations such as AMD may occur largely independently of NLRP3, NLRC4 or AIM2 sensor proteins. Alternatively, it is possible that multiple inflammasome sensor proteins are activated simultaneously and that inhibiting a single inflammasome sensor protein has no major effect on dampening the immune response. Overall, we demonstrate that protease enzyme CASP-1 is likely responsible for propagating inflammation and consequent photoreceptor cell death in the retina, and highlight how targeting of this downstream inflammasome component could offer more therapeutic promise than targeting individual inflammasome sensors.

Inflammasome-mediated inflammation and cell death are widely accepted to be associated with both wet and dry forms of AMD^1,3,17,23,33,34,51^, with *in vivo* inhibition of inflammasome-activated pro-inflammatory cytokine IL-1β shown to greatly improve the survivability of the retina following photo-oxidative damage^14^. However, while much of the current literature has focused on investigating the role of inflammasome sensor receptor NLRP3 in propagating this damage, little work has been done on other components of inflammasome pathways, in particular on the role of pro-inflammatory-cleavage protease CASP-1. Results from this study suggest that CASP-1-dependent inflammasomes are responsible for the propagation of inflammation and cell death in photo-oxidative damage-induced retinal degenerations.

This hypothesis is supported by other studies, in which the expression of *Casp-1* is upregulated following short term (1–24 hours), moderate bright white/blue light exposure in several *in vivo* mouse models^40–42^. These studies demonstrate that significant upregulation of *Casp-1* is correlated with increased levels of photoreceptor cell death^41,42^. Furthermore, CASP-1 inhibition or ablation in mice was shown to decrease levels of *Il-1β*, inflammation and subsequent retinal capillary degeneration in a model of diabetic retinopathy^52^, as well as reduced photoreceptor cell death in a model of retinitis pigmentosa^53^. In addition, more recently, Young *et al*., 2019, have shown that eyes pre-treated with an sGFP-TatCARD vector, that is, an intravitreally injected gene-delivered *Casp-1* inhibitor, were protected from sodium-iodate induced retinal degeneration^54^. Given the reduction in both ERG function and outer segment photoreceptor length seen in dim-reared *Casp1/11*^*−/−*^ mice compared to WT controls, it cannot be ruled out that a developmental difference resulting in reduced OS surface area could afford *Casp1/11*^*−/−*^ mice a level of protection against photo-oxidative damage. However, taking together the results of this study, with previous findings that demonstrate increased protection against degeneration using Casp-1 inhibitors in the retina^52–54^,and CNS^55,56^, it appears highly probable that CASP-1-dependent pathways play an important role in mediating inflammatory retinal cell death.

Due to the close genomic location of CASP-1 and CASP-11, the strain of knock-out mouse used *Casp1/11*^*−/−*^ is deficient in both the canonical and non-canonical Caspases, 1 and 11 respectively^43^. However, no change in retinal function or histology was seen in *Casp11*^*−/−*^ compared to WT controls, indicating that this pathway is most likely not involved in retinal degeneration. As CASP-11 is activated by LPS from gram-negative bacteria^57,58^, and the retina is immune privileged, this result is not unexpected. These results further support a role of CASP-1 dependent inflammasomes in the progression of retinal degeneration. We suggest that in retinal degenerations, CASP-1 propagates retinal inflammation and photoreceptor cell death via the cleavage and activation of pro-inflammatory cytokine IL-1β as well as possibly through its role in Gasdermin D pyroptotic pore formation. Further research into the role of both Gasdermin D and pyroptosis in the progression of retinal degenerations however, is necessary, in addition to exploring the therapeutic targeting of CASP-1.

NLRP3 is the best characterised CASP-1-dependent inflammasome sensor protein, and although widely studied, its role in the progression of retinal degenerations such as AMD has yet to be fully elucidated. The expression of NLRP3 has been reported in the lesion site in both the RPE and drusen of wet- and dry-AMD patients compared to healthy age-matched controls^29,33^, however, it is unclear whether this is a cause or consequence of AMD itself. In addition, research by Doyle *et al*., 2012 indicates that NLRP3 may play a protective or homeostatic role in wet-AMD pathogenesis^21^, showing using a laser-induced mouse model of CNV, that compared to WT controls, *Nlrp3*^*−/−*^ mice displayed increased CNV development and subretinal hemorrhage as well as increased macrophage infiltration to lesion areas^21^. In contrast to this investigation however, the role of NLRP3 in dry-AMD pathogenesis by the same group was only investigated in terms of capability to become activated by drusen component C1q, but not whether this actually occurred *in vivo*^21^. Furthermore, studies by Marneros *et al*. 2013 and 2016, demonstrate that *Nlrp3*^*−/−*^ mice crossed with a VEGF-A^hyper^ strain (a strain known to exhibit age-dependent features of both wet- and dry-AMD), showed reduced numbers of CNV lesions compared to controls, however, did not exhibit any protection against RPE degeneration or macrophage infiltration^35,36^. It therefore still remains unclear how NLRP3 is activated specifically in dry-AMD and more importantly if it plays a role at all.

To date, much of the research on uncovering the role of NLRP3 in AMD pathogenesis has investigated the mode of activation of NLRP3 in cell culture-based studies, primarily focusing on the RPE^19,21,22,27,28,31,32^. However, a recent review by Kosmidou *et al*.^30^ calls into question the specificity of cited NLRP3 antibodies, demonstrating that NLRP3 localisation in the retina could not be replicated^30^. *In situ* hybridisation results from this study detected *Nlrp3* labelling in the both the INL and GCL of mouse retinas at 5 days photo-oxidative damage and in human AMD retinas, a finding which is supported in another study using RNAscope *in situ* hybridization^37^. In addition, a recent publication has reported the expression of NLRP3 protein in both the GCL and nerve fiber layers of WT mice^59^. Taken together, the expression pattern of *Nlrp3* mRNA, and possibly protein, appears to reside in these neuronal cellular layers, suggesting *Nlrp3* is produced by these cell types. Our *in vitro* results further support an inner retina *Nlrp3* localisation, with *Nlrp3* expression shown in cell types located in the INL and GCL; MIO-M1 Müller like cells, C8B4 microglial-like cells as well as primary retinal microglia, with *Nlrp3* gene expression highly inducible in both microglial cell types following inflammasome stimulations. Regardless, the inner retinal localization and upregulation of *Nlrp3* demonstrated in this, and other works^37,59^, does not easily support an outer retina photoreceptor degeneration phenotype as seen in this model, and in AMD pathogenesis, additionally supporting the hypothesis that NLRP3 may not largely contribute to retinal degenerations. Further co-localisation studies are required however, to further identify unequivocally which retinal cells express *Nlrp3*, and if the transcript produced in these cells is subsequently packaged and exported to the RPE where the protein is widely reported to be expressed.

While proof-of-concept studies and RPE-based cell culture experiments appear to be common in the literature^17,21,24–29,60^, there exists a lack of *in vivo* studies that investigate the role of NLRP3 in AMD pathogenesis. For this reason, this study used a well-established photo-oxidative damage mouse model that recapitulates many important facets of dry-AMD pathogenesis including the upregulation of inflammatory pathways^14,46,48,61,62^. Results from this study indicate that while retinal *Nlrp3* gene expression increased over time in photo-oxidative damage, and *Nlrp3*^*−/−*^ mice had better-preserved retinal function following photo-oxidative damage; there was no change in NLRP3 protein expression, and mice injected with both NLRP3 inhibitor and siRNA showed unchanged or reduced retinal function and photoreceptor survivability. It is possible however, that the low-level expression changes in *Nlrp3* are able to be detected using sensitive techniques such as qPCR and *in situ* hybridisation, however, not by western blots.

MCC950 is a small selective inhibitor of NLRP3^63,64^ that has been shown to reduce levels of cleaved CASP-1 and IL-1β in response to both NLRP3 specific stimulation *in vitro*^64^, as well as in NLRP3-driven inflammatory diseases^65–68^. While the use of MCC950 has been investigated in a cell culture model of diabetic retinopathy^68^, and in RPE cell culture models of AMD^69^, NLRP3 inhibition via MCC950 had not been studied before in the retina. Further investigations are therefore still necessary to determine the bioavailability and clearance rate of this drug in the eye, as well as to test different dosages and methods of drug delivery. These results however, supported with significantly reduced *Nlrp3* gene expression 5 days after intravitreal injection with *Nlrp3* siRNA, suggest that NLRP3 may not play a key role in inflammasome-mediated photoreceptor cell death in the retina, and may potentially be upregulated as a consequence, rather than cause of degeneration, as previously questioned^36^.

Alternatively, like suggested by Doyle *et al*. 2012, NLRP3 could play a homeostatic or protective role in the retina. It is unclear exactly why mice deficient in NLRP3 but not NLRP3-inhibited mice have better-preserved retinal responses following photo-oxidative damage, however, we hypothesise that as dim-reared *Nlrp3*^*−/−*^ mice had reduced retinal function compared to WT dim-reared controls, that NLRP3 may play a role in normal retinal functioning, homeostasis, or in development; and that mice deficient in NLRP3 from birth may be less susceptible to inflammatory cell death than WT controls. Regardless, at the doses investigated and using intravitreal injection methods, NLRP3 inhibition did not appear to reduce photoreceptor cell death, inflammation or improve retinal function following photo-oxidative damage, suggesting that targeting NLRP3 may not slow the progression of retinal degenerations.

In addition to NLRP3, the role of alternative CASP-1-dependent inflammasome components in retinal degenerations was also investigated. ASC plays a critical role in both the NLRP3 and AIM2 inflammasome pathways, acting to bridge the activated PRR to the protease CASP-1^17^. Little has been reported on ASC in the literature, specifically in relation to retinal degenerations, however, what is clear is that from gene expression studies, *Asc* gene up-regulation can be mirrored to that of *Casp-1* and *Il-1β*, along with cell death^70^. What was unexpected however, was that unlike in *Casp1/11*^*−/−*^ mice, *Asc*^*−/−*^ mice, for both dim-reared and photo-oxidative damage conditions, had significantly lower ERG responses for a- and b-wave measures. It is unclear at this stage why this may be occurring; however, it is evident that the retina under normal or developing conditions requires the presence of functional ASC. A decreased retinal function was also observed for *Nlrp3*^*−/−*^ and *Casp1/11*^*−/−*^ mice under dim-reared conditions, suggesting that this inflammasome complex may be required for photoreceptor development, but not in photo-oxidative damage induced-degeneration. Further investigations are required to elucidate the function of ASC in normal retinal health as well as in diseased state.

Finally, in addition to investigations into NLRP3, the role of NLRC4 and AIM2 inflammasome sensor proteins in retinal degenerations was examined. As both of these inflammasome pathways have known roles in sensing and responding to intracellular pathogens including bacteria and viruses^71,72^, contribution to retinal degenerations following photo-oxidative damage was not expected. Although mice lacking NLRC4, AIM2 or ASC showed the same or higher levels of cell death than WT controls, a reduction in the total number of IBA-1^+^ microglia was seen. While an increased number of microglia is often correlated with an increased level of inflammation, and cell death, this is not a direct or causative relationship^73^. As microglia can exist in either a ramified/resting state; or an amoeboid/activated state, further investigations into the nature of the microglia in these inflammasome knock-out mice compared to WT controls needs to be investigated in the future. Given the reduced retinal function and increased cell death seen in *Aim2*^*−/−*^ and *Asc*^*−/−*^ mice compared to WT controls following photo-oxidative damage, it is possible that AIM2 sensor proteins, along with ASC, may play a protective role in the retina during degeneration induced by photo-oxidative damage.

While it is not clear which pathway(s) are mediating inflammation in the retina in AMD, overall results from this study demonstrate that the inflammasome protease enzyme CASP-1 may play a significant role in the propagation of inflammation and cell death that characterises many retinal degenerative diseases. Taken together, results from this work suggest that inflammatory cell death in retinal degenerative diseases may occur independently of NLRP3, or that there is functional redundancy between inflammasome sensor proteins. The targeting of molecular components downstream of inflammasome sensor proteins, in particular CASP-1, may provide more therapeutic potential, and will be the focus of future research directions.

## Methods

### Animal handling and photo-oxidative damage

All experiments were conducted in accordance with the ARVO Statement for the Use of Animals in Ophthalmic and Vision Research and with approval from the Australian National University’s (ANU) Animal Experimentation Ethics Committee (AEEC) (Ethics ID: A2017/41; Rodent models and treatments for retinal degenerations). Adult male and female C57BL/6J wild-type (WT), *Nlrp3*^*−/−*^, *Caspase11*^*−/−*^ *(Casp11*^*−/−*^*)*, *Caspase1/11*^*−/−*^ *(Casp1/11*^*−/−*^*)*, *Asc*^*−/−*^, *Nlrc4*^*−/−*^ and *Aim2*^*−/−*^ mice (aged between 60–90 postnatal days) were bred and reared under 12 h light/dark cycle conditions (5 lux) with free access to food and water. The C57BL/6J colony was genotyped for the presence of both the Rpe65^450M*et*^ polymorphism or the deleterious Crb1^rd8^ mutation using previously published primer sets^74,75^. Sequencing for these was conducted at the ACRF Biomolecular Resource Facility, ANU. All animals used possessed the Rpe65^450M*et*^ polymorphism but were free of the Crb1^rd8^ mutation. *Nlrp3*^*−/−*^, *Casp11*^*−/−*^ and *Casp1/11*^*−*/*−*^ strains were purchased from the Jackson Laboratory, while *Asc*^*−/−*^, *Nlrc4*^*−/−*^ and *Aim2*^*−/−*^ mice were supplied by Dr. Vishva M. Dixit (Genentech, CA, USA). The origin and characterization of mice strains have been previously described in detail^76–80^. Littermate age-matched WT and knock-out mice were randomly assigned to photo-oxidative damage (PD) and dim-reared control (DR) groups (N = 6–12 per group). Animals in the photo-oxidative damage group were continuously exposed to 100 K lux white LED light for a period of 1, 3, 5 or 7 days, as well as 14 days (7 days recovery post 7 days photo-oxidative damage) as described previously^46^, with the majority of experiments conducted for 5 days of photo-oxidative damage. Dim-reared, and 7 days recovery post-damage mice were maintained in 12 h light (5 lux)/dark cycle conditions.

In addition, adult (P60) Chemokine C-X3-C motif receptor 1; yellow fluorescent protein (Cx3cr1-cre YFP^+^) mice (APF, ANU) maintained on a C57BL6/J background were used to isolate primary retinal microglia. Animals were randomly assigned to dim-reared or photo-oxidative damage groups as above (N = 6).

### *In vivo* NLRP3 inhibition via intravitreal injection

*In vivo* protein and RNA interference was performed using NLRP3-specific inhibitor MCC950^64^ (Kindly gifted by Avril. A. B. Robertson) and *Nlrp3* Silencer® Select siRNA (Thermo Fisher Scientific, MA, USA), respectively. PBS and Silencer® Select negative control #1 siRNA were used as respective controls (Thermo Fisher Scientific, MA, USA). MCC950 was reconstituted in PBS at both 20 µM and 100 µM concentrations.

SiRNA was encapsulated using a cationic liposome-based formulation (Invivofectamine 3.0 Reagent; Thermo Fisher Scientific, MA, USA) as per the manufacturer’s instructions. To purify and increase the concentration of the siRNA formulation to a final concentration of 0.3 μg/μL in endotoxin-free PBS, the samples were spun at 4000 *g* through an Amicon Ultra-4 Centrifugal Filter Unit (Merck Millipore, Billerica, MA, USA). Intravitreal injections were performed as described in our previous publication^14^, 1 μL *Nlrp3* siRNA or negative control siRNA formulation was injected into both eyes of each C57BL/6J WT mouse. Following a half-day recovery, mice were then placed in photo-oxidative damage for the remainder of the 5 day paradigm. Mice were not pupil dilated (as described in our previous publication)^46^ on day one of photo-oxidative damage, to reduce irritation to the eye following intravitreal injection. Retinal RNA and whole eyes were collected following photo-oxidative damage as described previously^46^.

### Measurement of retinal function via electroretinography

Full-field scotopic electroretinography (ERG) was performed to assess the retinal function of dim-reared controls and animals after 5 days photo-oxidative damage, as well as injected mice as previously described^14^. Mice were dark-adapted overnight before being anaesthetised with an intraperitoneal injection of Ketamine (100 mg/kg; Troy Laboratories, NSW, Australia) and Xylazil (10 mg/kg; Troy Laboratories, NSW, Australia). Both pupils were dilated with one drop each of 2.5% w/v Phenylephrine hydrochloride and 1% w/v Tropicamide (Bausch and Lomb, NY, USA). A single- or twin-flash paradigm was used to elicit a mixed response from rods and cones, and an isolated cone response, respectively. Flash stimuli for mixed responses were provided by an LED-based system (FS-250A Enhanced Ganzfeld, Photometric Solutions International, VIC, AUS), over a stimulus intensity range of 6.3 log cd s m^−2^ (range −4.4–1.9 log cd s m^−2^). Amplitudes of the a-wave and b-wave were analysed using LabChart 8 software (AD Instruments, Dunedin, NZ) and data were expressed as the mean wave amplitude ± SEM (μV).

### Optical coherence tomography (OCT)

Cross-sectional images of live mouse retinas were taken 1 to the optic nerve using a Spectralis HRA + OCT device (Heidelberg Engineering, Heidelberg, Germany) as previously described^46^. Eye gel (GenTeal; Novartis, NSW, AUS) was administered to both eyes for recovery.

Using OCT cross-sectional retinal images or retinal cryosections, and ImageJ software (National Institutes of Health, Bethesda, MD, USA), outer nuclear layer (ONL) thickness was either calculated as the ratio of the thickness of the ONL to the whole retinal thickness (outer limiting membrane to the inner limiting membrane) for OCT images, or ONL thickness (μM) for retinal cryosections. The length (μM) of photoreceptor inner and outer segments (IS and OS) were also measured. ONL, IS and OS thickness was measured five times at 1-mm intervals across the retina (superior for IS and OS) and averaged. In addition, the thickness of the ONL was determined by counting the number of rows of nuclei (photoreceptor cell bodies) in the area of retinal lesion development (1 mm superior to the optic nerve head), to quantify photoreceptor survival. The process of ONL photoreceptor cell row quantification was performed five times per retina, on two retinal sections at comparable locations per mouse.

### Tissue collection and preparation

Animals were euthanised with CO_2_ following functional ERG analysis. The superior surface of the left eye from each animal was marked and enucleated, then immersed in 4% paraformaldehyde (PFA) for 3 hours. Eyes were then cryopreserved in 15% sucrose solution overnight, embedded in OCT medium (Tissue Tek, Sakura, JP) and cryosectioned at 12 μm in a parasagittal plane (superior to inferior) using a CM 1850 Cryostat (Leica). To ensure accurate comparisons were made for histological analysis, only sections containing the ON head were used for analysis (N = 6 per experimental group). The retina from the right eye of each mouse used was excised through a corneal incision and placed into Cellytic M buffer (Sigma-Aldrich, MO, USA) containing a Protease Inhibitor Cocktail (Sigma-Aldrich, MO, USA) to extract whole cell protein lysates and then stored at −80 °C until further use (N = 6 per experimental group). Some retinas were placed into RNAlater solution (Thermo Fisher Scientific, MA, USA) at 4 °C overnight and then stored at −80 °C until further use (N = 6 per experimental group).

### Cell culture

Murine photoreceptor-derived 661 W cells (kindly gifted by Dr. Muayyad R. Al-Ubaidi, Dept. of Cell Biology, University of Oklahoma Health Sciences Centre, Oklahoma City, OK, USA)^81^, murine brain derived microglia C8B4 (American Tissue Culture Collection (ATCC), Virginia, USA)^82^, immortalised human Müller-like MIO-M1 (Moorfield’s Institute of Ophthalmology, London, UK)^83^ and immortalised human RPE-like aRPE19 (ATCC)^84^ within five passages of authentication were used for these experiments. Cells were validated for species authenticity (CellBank, Sydney, AUS). These cells were cultured as previously published^85^, in growth medium containing Dulbecco’s Modified Eagle Medium (DMEM; Sigma-Aldrich) supplemented with 10% fetal bovine serum (FBS; Sigma-Aldrich, MO, USA), 6 mM L-glutamine (Thermo Fisher Scientific, MA, USA) and antibiotic-antimycotic (100U/ml penicillin, 100 μg/ml streptomycin; Thermo Fisher Scientific, MA, USA). Cells were maintained in dim conditions in a humidified atmosphere of 5% CO_2_ at 37 °C, and passaged by trypsinization every 3–4 days.

#### Fluorescence-activated cell sorting (FACS) of microglia

FACS was utilised to isolate Cx3cr1-YFP^+^ primary retinal microglia from 5 day photo-oxidative damage mice, as well as dim-reared controls. Briefly, retinas were pooled and collected in Hanks buffered saline solution (HBSS, Gibco; Thermo Fisher Scientific, MA, USA) and subsequently mechanically chopped up using scissors and digested in digestion solution (HBSS with 2.5 mg/mL papain (Worthington Biochemical, NJ, USA), 200U DNAse I (Roche Diagnostics, NSW, AUS) 5 μg/mL catalase (Sigma-Aldrich, MO, USA), 10 μg/mL gentamycin (Sigma-Aldrich, MO, USA) and 5 μg/mL superoxide dismutase (Worthington Biochemical, NJ, USA)) at 37 °C for 8 minutes, followed by 10 minutes at 8 °C. Following digestion, tissue suspensions were spun down at 1250 rpm of 5 minutes at 4 °C and then resuspended and neutralised in neutralisation buffer ((HBSS with 4% bovine serum albumin (BSA, Thermo Fisher, MA, USA), 50 μg/mL antipain dihydrochloride (Roche Diagnostics, NSW, AUS), 200U DNAse I (Roche Diagnostics, NSW, AUS) 5 μg/mL catalase (Sigma-Aldrich, MO, USA), 10 μg/mL gentamycin (Sigma-Aldrich, NSW, AUS) and 5 μg/mL superoxide dismutase (Worthington Biochemical, NJ, USA)) for 10 minutes on ice. Samples were re-spun, washed in 1x PBS (Gibco, Thermo Fisher Scientific, MA, USA), and resuspended in 1.5 mL of 1x PBS with 1% of 200 U/mL DNase I (Roche Diagnostics, NSW, AUS) and 0.5% MgCl_2._ Each solution was then filtered through 70μm MACS SmartStrainers (Miltenyi Biotec, Cologne, Germany) into a 5 ml tube. Cell populations were isolated by FACS (BD FACSMelody cell sorter, CAM, JCSMR) using BD FACSChorus software (BD Biosciences). Retinal microglia isolated were seeded into 24 well plates containing DMEM/F12 (Gibco, Thermo Fisher Scientific, MA, USA) supplemented with 2.5 ng/mL macrophage colony stimulating factor (M-CSF) (STEMCELL Technologies, VIC, AUS) and 0.5 ng/mL granulocyte macrophage colony stimulating factor GM-CSF (STEMCELL Technologies, VIC, AUS), and grown until confluency.

### *In vitro* NLRP3 inflammasome stimulation

Immortalised and primary cell cultures were either chemically stimulated or light damaged to establish *in vitro* inflammatory models. At passage two, cells were split and seeded into 24 well plates (at a density of 2.5 × 10^4^ cells per well for 661 W, 15 × 10^4^ cells per well for C8B4, 2.0 × 10^4^ cells per well for MIO-M1 and 10 × 10^4^ cells per well for aRPE19), and into 8-well chamber slides (5000 cells per well for all cell types) in growth medium, and were incubated overnight in dim conditions in a humidified atmosphere of 5% CO_2_ at 37 °C until confluency. 24 hours prior to stimulation, cells in the 24-well plate and 8-well chamber slides were incubated in reduced-serum DMEM (supplemented with 1% FBS, L-glutamine and antibiotic-antimycotic (Pen/Strep)) and after 24 hours were stimulated for NLRP3 activation.

#### *In vitro* photo-oxidative damage

661 W cells were exposed to 15,000 lux light (2.2 mW/cm^2^; irradiance measured with PM100D optical power meter, THORLABS, NJ, USA) from two white fluorescent lamps (2 × 10 W T4 tri-phosphor 6500 K daylight fluorescent tubes; Crompton, NSW, Australia), for 5 hours with 5% CO_2_ at 37 °C. For dim control cells, one plate and one chamber slide in the incubator were completely wrapped in aluminium foil to avoid light exposure. For air/gas exchange, six small incisions were cut on the aluminium foil. Following incubation, cells in both the dim and photo-oxidative damage chamber slides were washed in PBS before being fixed in 4% PFA for 2 hours at 4 °C and then were maintained in PBS at 4 °C until further use. Cells in each of the 24 wells were washed with PBS and were then triturated either in TRIzol (Thermo Fisher Scientific, MA, USA) and stored at −80 °C until further use (n = 6 per experimental group), or in placed into CellLytic M buffer (Sigma-Aldrich, MO, USA) containing a Protease Inhibitor Cocktail (Sigma-Aldrich, MO, USA) and then stored at −80 °C until further use (N = 6 per experimental group).

#### C8B4, Primary microglia, MIO-M1 and aRPE19

Immortalised and primary cells were stimulated using a number of previously published techniques. Microglia were primed with 20 ng/mL LPS from *Escherichia coli* 0111:B4 (N4391, Sigma Aldrich, MO, USA) for 4 hours and stimulated with either 5 mM ATP (A6419, Sigma Aldrich, MO, USA) for 0.5 hours or 10 μM Nigericin sodium salt from *Streptomyces hygroscopicus* (N7143, Sigma Aldrich, MO, USA) for 1 hour (C8B4 only)^86^. Immortalised Müller cells (MIO-M1) and RPE cells (aRPE-19) were primed with 20 ng/mL LPS for 4 hours, or either 10 ng/mL TNF-α (210-TA, R&D Systems, MN, USA) for 24 hours for MIO-M1 cells^29,87^, or 50 ng/mL Recombinant Human Interleukin-1α (IL-1α) Protein (ab9615, Abcam, Cambridge, UK) for 24 hours for aRPE19 cells^29,30,87^. MIO-M1 and aRPE-19 cells were then stimulated with 5 mM ATP for 0.5 hours following all priming agents. Unstimulated cells were used as negative controls.

### Immunolabelling

Immunohistochemical analysis of retinal cryosections was performed as previously described^62^. Immunocytochemistry was performed using a protocol previously described^85^. Haematoxylin and Eosin staining was performed by covering the retinal cryosections in 1x PBS for 10 minutes, followed by 3 minutes in 95% ethanol and 3 minutes in 70% ethanol. Following, slides were rinsed in MilliQ water and then stained with Harris Haematoxylin for 2 minutes (Sigma-Aldrich, MO, USA) before being rinsed again in MilliQ water. Finally, sections were counterstained with Eosin Y (Sigma-Aldrich, MO, USA) for 2.5 minutes, dehydrated in an each of 70%, 95% and 100% ethanol for 3 minutes each, rinsed in MilliQ and coverslipped. Details of primary antibodies used are displayed in Table 1. Fluorescence was visualised and images obtained using a laser-scanning A1^+^ confocal microscope (Nikon, Tokyo, Japan). Images panels were analysed using ImageJ (NIH, MD, USA) and assembled using Photoshop CS6 software (Adobe Systems, CA, USA).

**Table 1:** List of primary antibodies used for immunolabelling.

Antibody	Dilution	Company	Catalogue number
Rabbit anti IBA-1	1:500	Wako, Osaka, JP	019–19741
Mouse anti NLRP3	1:100	AdipoGen Life Sciences, Liestal, Switzerland.	AG-20B-0014-C100
Mouse anti Caspase-1	1:200	AdipoGen Life Sciences, Liestal, Switzerland.	AG-20B-0042-C100
Goat anti IL-1β/IL-1F2	1:100	R&D systems, MN, USA	AF-401-NA
Harris Haematoxylin	—	Sigma-Aldrich, MO, USA	517-28-2
Eosin Y	—	Sigma-Aldrich, MO, USA	15086-94-9

For IBA-1 immunohistochemistry, the number of IBA-1^+^ cells (a marker of retinal microglia and macrophages) was counted across the superior and inferior retina in retinal cryosections. This quantification was performed on two retinal sections per mouse and averaged. Retinal cryosections were stained with the DNA-specific dye bisbenzimide (1:10000, Sigma-Aldrich, MO, USA) to visualise the cellular layers.

### *In situ* hybridisation (ISH)

To localise *Nlrp3* messenger RNA (mRNA) transcripts in mouse retinal and human AMD donor retinal tissue cryosections, riboprobes specific for mouse *Nlrp3* and *Rhodopsin* were developed (Thermo Fisher Scientific, MA, USA). Primers were designed specific to each gene (Table 2) and a T7 Polymerase tag was added to the 5’ end of the reverse primer in each set. cDNA was amplified using PCR (Veriti 96 well Thermal Cycler, Applied Biosystems; Thermo Fisher Scientific, MA, USA), and verified on a 1% agarose gel (Sigma Aldrich, MO, USA). PCR products were subsequently purified using ammonium acetate precipitation. Briefly, ammonium acetate (Sigma Aldrich, MO, USA) was added at ¼ volume to PCR product, following which ice-cold 100% ethanol was added at 10x the volume of ammonium acetate. Samples were centrifuged at 13,000 rpm for 15 minutes at 4 °C and supernatant was removed and replaced with 70% ice-cold ethanol to wash pellet. Following a 2 min spin at the same settings, supernatant was again removed and replaced with Ultrapure water (Gibco, Thermo Fisher Scientific, MA, USA) for elution. Purified probe templates were subsequently quantified using ND-1000 spectrophotometer (Nanodrop Technologies, DE, USA) for RNA yield.

**Table 2:** Primer sequences for *in situ* hybridisation riboprobe development.

Gene	Primer sequences	Temp °C
*Rhodopsin*	Forward: GAACTGTATGCTCACCA *Reverse:* ATATATTAATACGACTCACTATAGG*GACATCCATGTTCCCTT	Hyb: 58 Post Hyb: 60
*Nlrp3*	Forward: ACCTCAACAGTCGCTACACG *Reverse:* ATATATTAATACGACTCACTATAGG*TAGACTCCTTGGCGTCCTGA	Hyb: 56 Post Hyb: 60

*Italised portion on reverse primer is T7 Polymerase sequence.

These purified probe templates were then synthesised into a digoxigenin (DIG)- labeled riboprobe that was specific to mouse *Nlrp3* or *Rhodopsin*, run as a positive control, according to our previously published protocol^88^.

Mouse dim-reared and 5 day photo-oxidative damage retinal sections used were prepared as above. Adult human eyes were collected with informed consent following the tenets of the Declaration of Helsinki, through the Lions NSW Eye Bank, (NSW, Australia) with ethical approval from the Human Research Ethics Committee of both the University of Sydney and The Australian National University (Project No. 2012/218). Grading of the eyes was performed by experienced graders according to published pathological criteria^89^, and ranged from normal to early- or late- dry AMD. Human AMD tissue was catalogued and processed for sectioning based on previously published methods^90^.

ISH was performed using established protocols^91^. In brief, both human and mouse riboprobes (Table 2) were hybridised overnight at 56 °C and then washed in saline sodium citrate (p.H 7.4) at 60 °C. the bound probe was subsequently visualised using nitro blue tetrazolium and 5-bromo-4-chloro-3-indolyl phosphate (NBT/BCIP) (Sigma Aldrich, MO, USA).

### TUNEL staining and quantification

Terminal deoxynucleotidyl transferase (Tdt) dUTP nick end labeling (TUNEL), was used as a measure of photoreceptor cell death. TUNEL *in situ* labelling was performed on retinal cryosections using a Tdt enzyme (Cat# 3333566001, Sigma-Aldrich, MO, USA) and biotinylated deoxyuridine triphosphate (dUTP) (Cat# 11093070910, Sigma-Aldrich, MO, USA) as previously described^92^. In each retinal section, the total number of TUNEL^+^ cells were counted including both the superior and inferior retina. This process of quantification was performed on two retinal sections per animal, and was calculated as the average number of TUNEL^+^ cells per retinal section. Images of TUNEL staining were captured with the A1^+^ confocal microscope at 20× magnification.

### Quantitative real-time polymerase chain reaction

Total RNA was extracted from the retinal and cell samples as described previously^61^, using a combination of TRIzol (Thermo Fisher Scientific, MA, USA) and an RNAqueous Micro Total RNA Isolation kit (Thermo Fisher Scientific, MA, USA). The concentration and purity of each RNA sample was assessed using the ND-1000 spectrophotometer (Nanodrop Technologies, DE, USA).

Following purification of RNA, cDNA was synthesised from 1 µg of each RNA sample using a Tetro cDNA Synthesis Kit (Bioline, London, UK) according to the manufacturer’s protocol. Gene expression was measured using qRT-PCR using both mouse and human specific TaqMan hydrolysis probes (Thermo Fisher Scientific, MA, USA), as shown in Table 3. The TaqMan probes, cDNA and TaqMan Gene Expression Master Mix (Thermo Fisher Scientific, MA, USA) were plated in a 384-well transparent plate. Each reaction was performed in technical duplicate and was carried out using a QuantStudio 12 K Flex RT-PCR machine (Thermo Fisher Scientific, MA, USA). Analysis was performed using the comparative C_t_ method (ΔΔC_t_). Results were analysed as a percent change relative to control samples, and normalised to reference gene glyceraldehyde-3-phosphate dehydrogenase (*Gapdh*).

**Table 3:** TaqMan hydrolysis probes (Thermo Fisher Scientific, MA, USA) used for qRT-PCR.

Gene symbols	Gene name	Catalogue number
*Asc/Pycard*	PYD and CARD domain containing	Mm00445747_g1
*Casp-1*	Caspase-1	Mm00438023_m1 Hs00354836_m1
*Gapdh*	Glyceraldehyde-3-Phosphatase Dehydrogenase	Mm01536933_m1 Hs02786624_g1
*Il-1β*	Interleukin-1β	Mm00434228_m1 Hs01555410_m1
*Il-18*	Interleukin-18	Mm00434226_m1
*Nlrp3*	NLR family pyrin domain containing 3	Mm00840904_m1 Hs00918082_m1

### Western blot

Western blot was used to measure the protein expression of NLRP3, CASP-1 and IL-1β in retinas from dim-reared and photo-oxidative damage WT, *Nlrp3*^*−*/*−*^, and *Casp1/11*^*−/−*^ mice. The blot was performed using whole retinal protein lysates according to previously described methods^50^. 20 µg of denatured protein was loaded into a 4–20% Mini-Protean TGX Precast Protein gel (Bio-Rad, CA, USA) followed by semi-dry transfer to a PVDF membrane. Primary and secondary antibodies used are detailed in Table 4. The protein was visualised with chemiluminescence using a Clarity Western ECL kit (Bio-Rad, CA, USA) and images were captured and analysed using a Chemidoc MP with Image Lab software (Bio-Rad, CA, USA).

**Table 4:** List of antibodies used for Western Blot.

Antibody	Dilution	Company	Catalogue number
Mouse anti NLRP3	1:1000	AdipoGen Life Sciences, Liestal, Switzerland.	AG-20B-0014-C100
Mouse anti Caspase-1	1:1000	AdipoGen Life Sciences, Liestal, Switzerland.	AG-20B-0042-C100
Goat anti IL-1β/IL-1F2	1:1000	R&D systems, MN, USA	AF-401-NA
Rabbit anti GAPDH	1:3000	Sigma Aldrich, MO, USA	G9545
Goat anti Rabbit Horseradish peroxidase (*HRP)* conjugate	1:1000	Bio-rad, CA, USA	170–6515
Goat anti mouse Horseradish peroxidase (*HRP)* conjugate	1:1000	Bio-rad, CA, USA	170–6516
Chicken anti goat IgG (H + L) secondary antibody HRP	1:3000	Thermo Fisher Scientific, MA, USA	A15963

### IL-1β Enzyme-linked immunosorbent assay (ELISA)

IL-1β levels in WT and KO mouse whole retinal protein extracts were determined using IL-1β ELISA (ELISAkit.com, Scoresby, Australia) according to the manufacturer’s instructions.

### Mouse cytokine/chemokine magnetic bead assay

Cytokine/chemokine levels in WT and KO mouse whole retinal protein extracts were determined using a multiplex assay for IL-1β, IL-6 and CXCL1 according to the manufacturer’s instructions. (Cat# MCYTOMAG-70K, Merck Millipore, MA, USA).

### Statistical analysis

All graphing and statistical analysis was performed using Prism 6 (GraphPad Software, CA, USA). An unpaired Student *t* test, one-way analysis of variance (ANOVA), or two-way ANOVA with Tukey’s multiple comparison post-test were utilised to determine the statistical outcome; a *P* value of < 0.05 was considered statistically significant. All data was expressed as the mean ± SEM.

## Supplementary information

supplementary information.
