# Supplementary material for: Caspase-1-dependent inflammasomes mediate photoreceptor cell death in photo-oxidative damage-induced retinal degeneration
Source: Sci Rep. 2020 Feb 10;10:2263. doi: 10.1038/s41598-020-58849-z (PMC7010818; doi:10.1038/s41598-020-58849-z)
Supplement: Supplementary file 1 — supplementary information. [file 41598_2020_58849_MOESM1_ESM.docx]

**Caspase-1-dependent inflammasomes mediate photoreceptor cell death in photo-oxidative damage-induced retinal degeneration.**

Yvette Wooff^a,b^, Nilisha Fernando^a^. Josephine H.C. Wong^a^, Catherine Dietrich^a^, Riemke Aggio-Bruce^a^, Joshua A. Chu-Tan^a,b^, Avril A. B. Robertson^c^, Sarah L. Doyle^d^, Si Ming Man^a^, Riccardo Natoli^a,b*^.

^a^ The John Curtin School of Medical Research, The Australian National University, Canberra, ACT, Australia.

^b^ The ANU Medical School, The Australian National University, Canberra, ACT, Australia.

^c^ School of Chemistry and Molecular Bioscience, The University of Queensland, St. Lucia, QLD 4072, Australia.

^d^ Department of Clinical Medicine, School of Medicine, Trinity College Institute of Neuroscience, Trinity College Dublin, Dublin 2, Ireland.

*corresponding author

Email: Riccardo.natoli@anu.edu.au

**
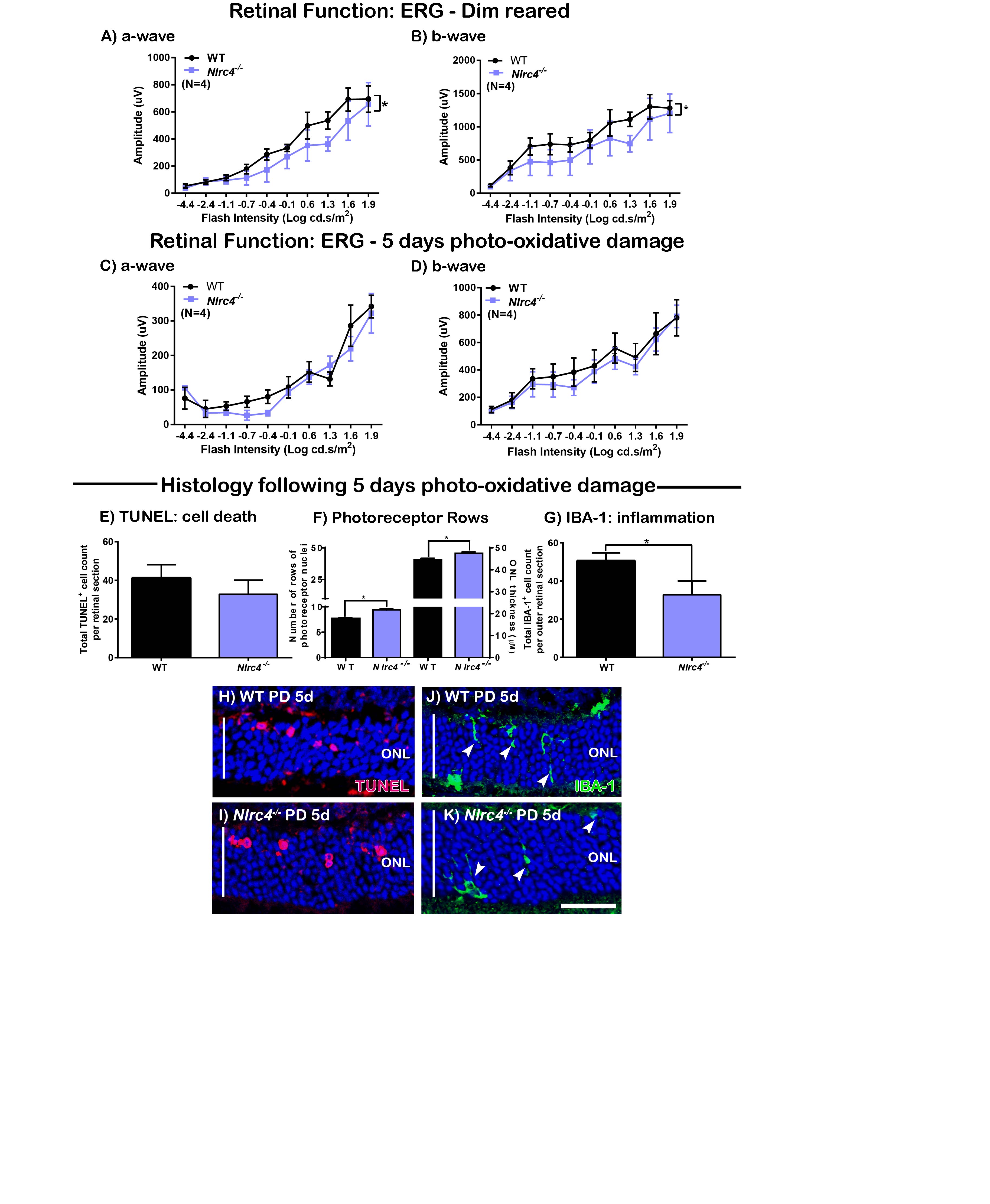
**

**Supplementary Figure 1. *Nlrc4^-/-^* mice have unchanged retinal function and inflammation compared to WT controls.**

**A-D** Retinal function was measured before (DR) and after 5 days PD using ERG. *Nlrc4^-/-^* mice had significantly reduced function compared to WT DR controls for both (A) a-wave or (B) b-wave responses (P<0.05, N=4), however there was no significant change in function in *Nlrc4^-/-^* for (C) a-wave and (D) b-wave measures following 5 days PD compared to WT controls (P<0.05, N=4). **E-G** The effect of *Nlrc4* deficiency on photoreceptor cell death and inflammation following 5 days PD. (E) No change was seen in TUNEL^+^ cell counts in the ONL (P>0.05, N=4), however, *Nlrc4^-/-^* mice had (F) an increased number of photoreceptor cell rows and an increased ONL thickness compared to WT controls (P<0.05, N=4). (G) *Nlrc4^-/-^ mice* had decreased total numbers of IBA-1^+^ cells in the outer retina compared to WT PD controls (P<0.05, N=4). **H-K** Representative confocal images showing an unchanged number of TUNEL^+^ cells and increased number of photoreceptor rows in (H) WT PD mice compared to (I) *Nlrc4^-/-^* mice, as well as an increased number of IBA-1^+^ cells in the outer retina of (J) WT mice compared to (K) *Nlrc4^-/-^* mice following 5 days PD. Scale bars = 25μM.


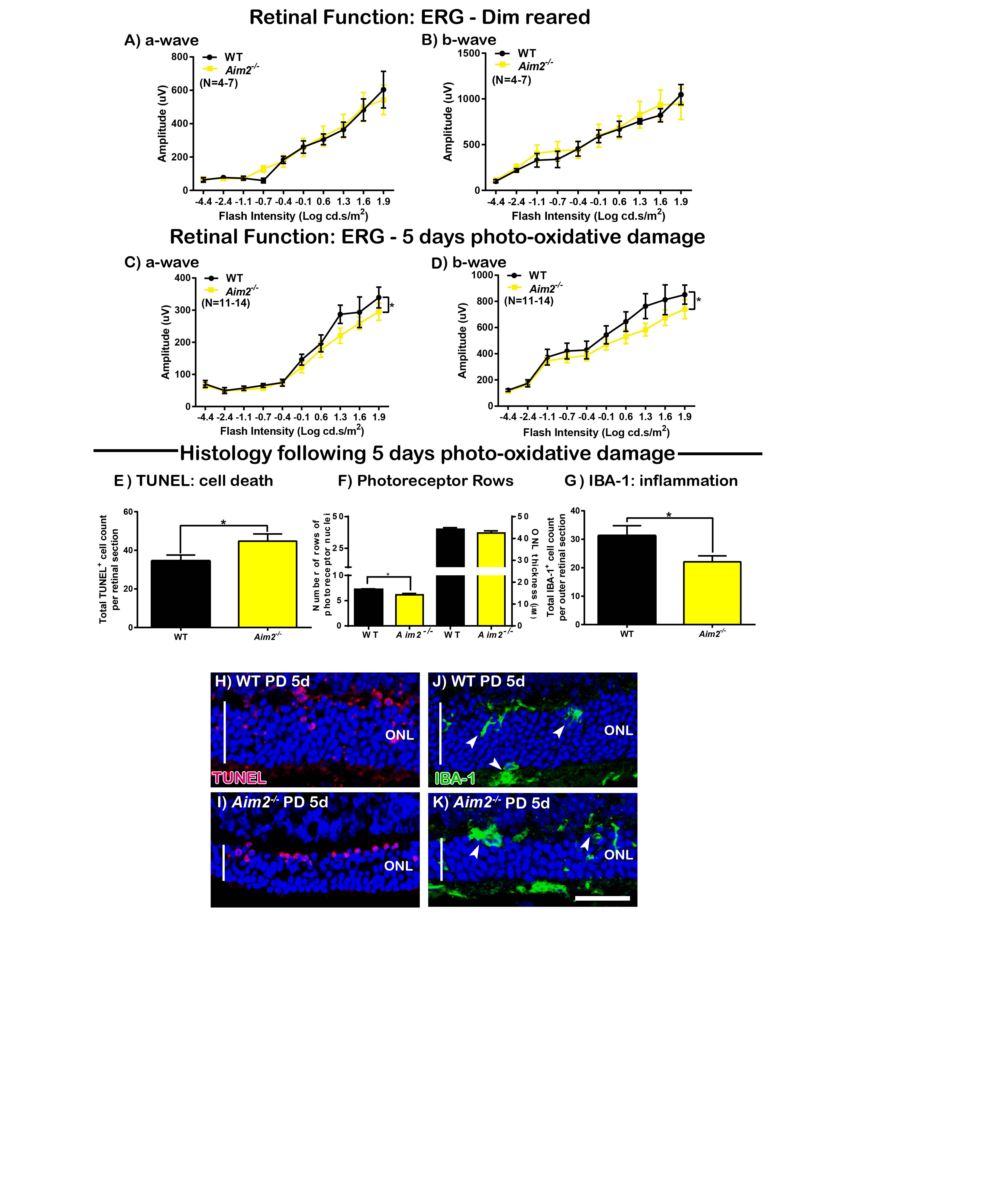


**Supplementary Figure 2.** ***Aim2*^-/-^ mice have reduced retinal cell function and increased cell death in response to photo-oxidative damage.**

**A-D** Retinal function was measured before (DR) and after 5 days PD using ERG. No significant difference was shown in DR *Aim2^-/-^* mice compared to WT controls for either (A) a-wave or (B) b-wave responses (P>0.05, N=4-7). *Aim2^-/-^* mice however had significantly decreased retinal function for (C) a-wave and (D) b-wave measures following 5 days PD compared to WT controls (P<0.05, N=11-14). **E-G** The effect of *Aim2* deficiency on photoreceptor cell death and inflammation following 5 days PD. *Aim2^-/-^* mice had increased levels of photoreceptor cell death as shown by significantly (E) increased TUNEL^+^ cell in the ONL, and (F) a decreased number of photoreceptor cell rows, compared to WT controls (P<0.05, N=11-14). No significant difference was seen in ONL thickness measurements however, between WT and *Aim2^-/-^* mice (P>0.05, N=11-14). (G) *Aim2* deficiency however resulted in reduced numbers of IBA-1^+^ cells in the outer retina compared to WT PD controls (P<0.05, N=11-14). **H-K** Representative confocal images showing a decreased number of TUNEL^+^ cells and increased number of photoreceptor rows in (H) WT PD mice compared to (I) *Aim2^-/-^* mice, as well as an increased number of IBA-1^+^ cells in the outer retina of (J) WT mice compared to (K) *Aim2^-/-^* mice following 5 days PD. Scale bars = 25μM.

**
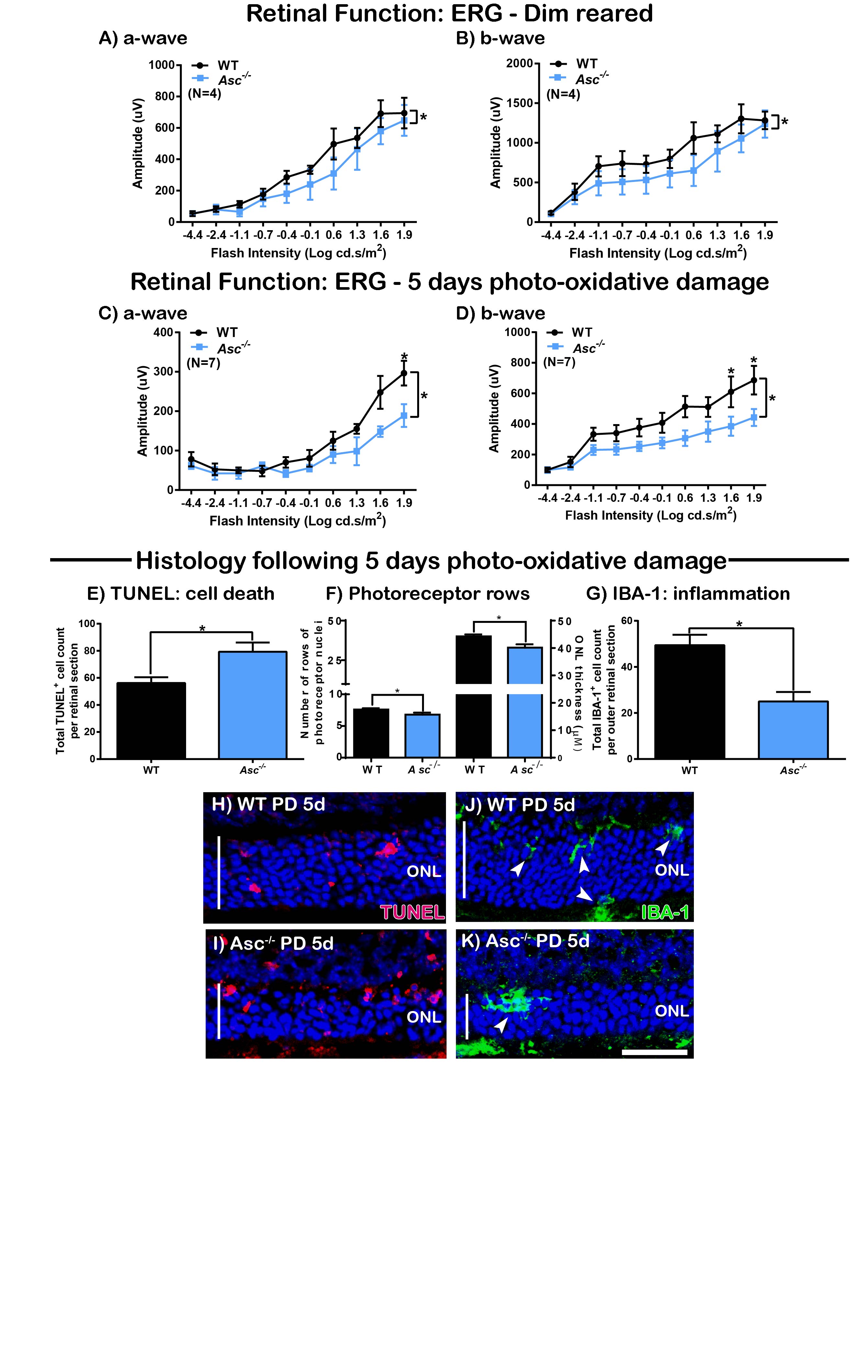
**

**Supplementary Figure 3.** ***Asc*^-/-^ mice have reduced retinal function and increased cell death in response to photo-oxidative damage.**

**A-D** Retinal function was measured before (DR) and after 5 days PD using ERG. *Asc^-/-^* mice had significantly lower retinal function in DR conditions compared to WT controls, for both (A) a-wave and (B) b-wave measures (P<0.05, N=4). In addition, following PD, *Asc^-/-^* mice had significantly lower retinal function than WT PD controls for both (C) a-wave and (D) b-wave responses (P<0.05, N=4). **E-G** The effect of *Asc* deficiency on photoreceptor cell death and inflammation following 5 days PD. *Asc^-/-^* mice had increased levels of photoreceptor cell death as shown by significantly (E) increased TUNEL^+^ cell in the ONL, and (F) a decreased number of photoreceptor cell rows and ONL thickness, compared to WT controls (P<0.05, N=4). (G) *Asc^-/-^ mice* had significantly reduced numbers of IBA-1^+^ cells in the outer retina compared to WT PD controls (P<0.05, N=4). **H-K** Representative confocal images showing a decreased number of TUNEL^+^ cells and increased number of photoreceptor rows in (H) WT PD mice compared to (I) *Asc^-/-^* mice, as well as an increased number of IBA-1^+^ cells in the outer retina of (J) WT mice compared to (K) *Asc^-/-^* mice following 5 days PD. Scale bars = 25μM.


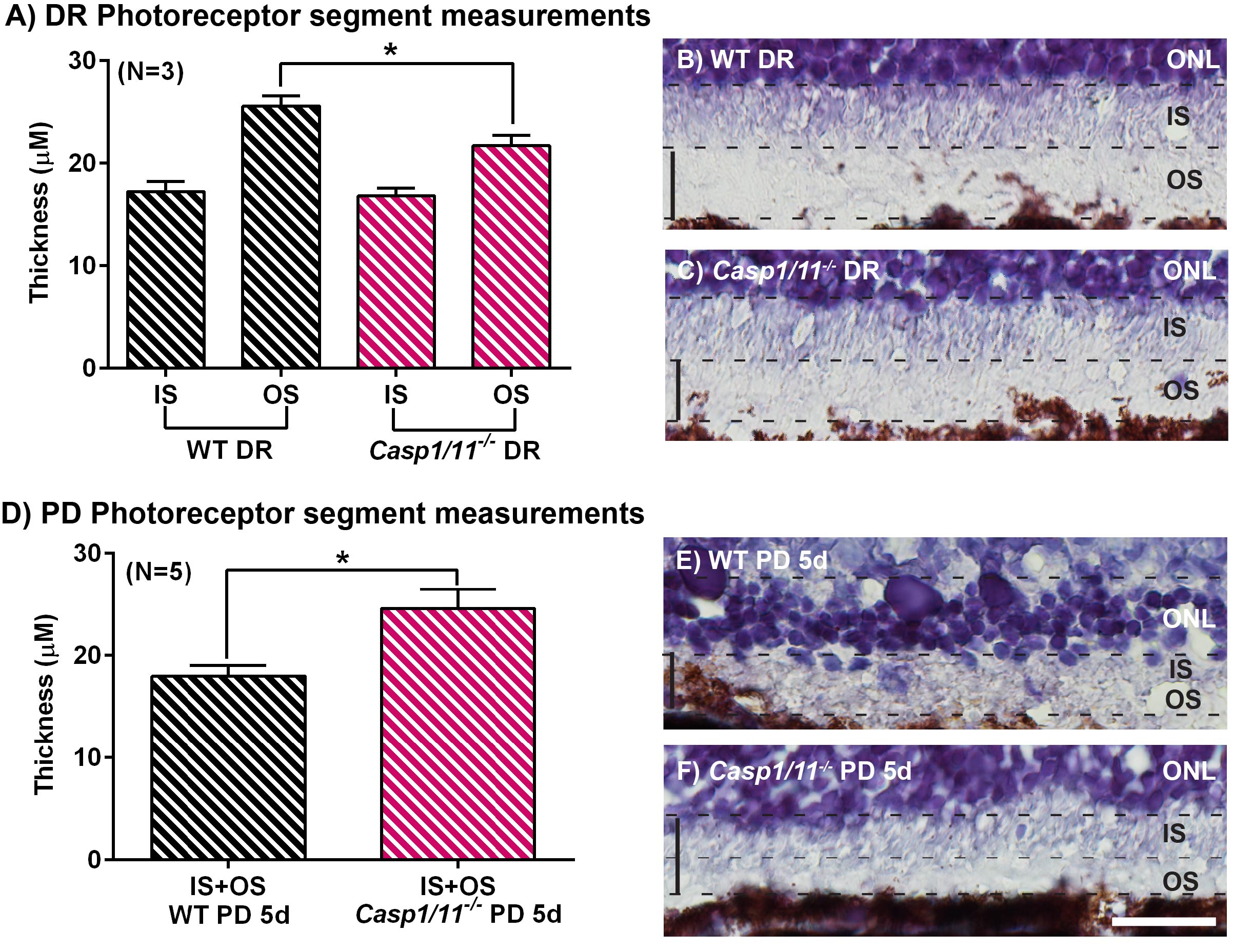


**Supplementary Figure 4: CASP-1 may be required for normal photoreceptor development in the mouse retina.**

**A-C** The effect of CASP-1 on photoreceptor segment length in normal conditions. Thickness measurements of the inner (IS) and outer segment (OS) of WT and *Casp1/11^-/-^* DR mice show no significant change in IS length, but a significant reduction in the OS length of *Casp1/11^-/-^* mice compared to WT controls (A). Representative bright-field images show significantly increased OS length in the retina of WT DR mice (B), compared to *Casp1/11^-/-^* DR mice (C). **D-F** The effect of CASP-1 on photoreceptor segment length following 5 days PD. Thickness measurements of the combined photoreceptor IS and OS of WT and *Casp1/11^-/-^* PD mice show a significant reduction in the OS length of WT mice compared to *Casp1/11^-/-^* PD mice (D). Representative bright-field images show significantly shorter OS length in the retina of WT PD mice (E), compared to *Casp1/11^-/-^* PD mice (F). Scale bars = 25μM.


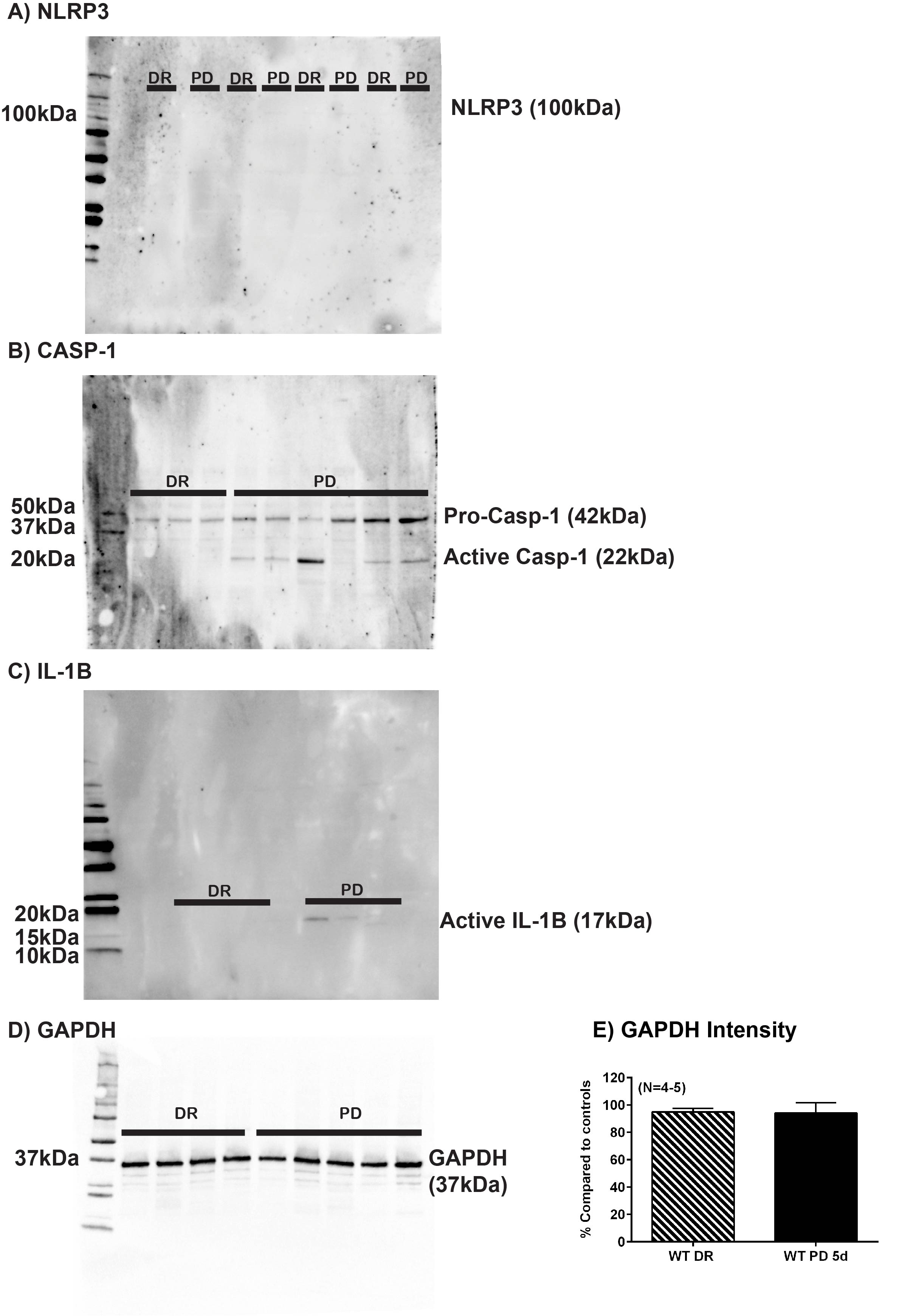


**Supplementary Figure 5: Western Blots.**

**A-C** Representative full-length western blots showing expression of inflammasome proteins in dim-reared and photo-oxidative damaged retinas. NLRP3 (100kDa) protein expression was weak, and unchanged between dim-reared and 5 day photo-oxidative damaged mice (A). Dim-reared mice showed expression of pro-CASP-1 (42kDa), however no expression of active-CASP-1. In addition, active-CASP-1 expression (22kDa) was seen in photo-oxidative damaged mice retinas (B). No expression of IL-1B (17kDa) was seen in retinas of dim-reared mice, however was seen in retinas of mice exposed to 5 days photo-oxidative damage (C). Unchanged GAPDH (37kDa) protein levels between dim-reared and 5 days photo-oxidative damage retinas (D and E).
